# Host-glycan metabolism is regulated by a species-conserved two-component system in *Streptococcus pneumoniae*

**DOI:** 10.1371/journal.ppat.1008332

**Published:** 2020-03-04

**Authors:** Patrick Rosendahl Andreassen, Claudia Trappetti, Vikrant Minhas, Flemming Damgaard Nielsen, Kevin Pakula, James C. Paton, Mikkel Girke Jørgensen

**Affiliations:** 1 Department of Biochemistry and Molecular Biology, University of Southern Denmark, Odense, Denmark; 2 Department of Molecular and Biomedical Science, University of Adelaide, Adelaide, Australia; The University of Alabama at Birmingham, UNITED STATES

## Abstract

Pathogens of the *Streptococcus* genus inhabit many different environmental niches during the course of an infection in a human host and the bacteria must adjust their metabolism according to available nutrients. Despite their lack of the citric-acid cycle, some streptococci proliferate in niches devoid of a readily available carbohydrate source. Instead they rely on carbohydrate scavenging for energy acquisition, which are obtained from the host. Here we discover a two-component system (TCS07) of *Streptococcus pneumoniae* that responds to glycoconjugated structures on proteins present on the host cells. Using next-generation RNA sequencing we find that the uncharacterized TCS07 regulon encodes proteins important for host-glycan processing and transporters of the released glycans, as well as intracellular carbohydrate catabolizing enzymes. We find that a functional TCS07 allele is required for growth on the glycoconjugated model protein fetuin. Consistently, we see a TCS07-dependent activation of the glycan degradation pathway. Thus, we pinpoint the molecular constituents responsible for sensing host derived glycans and link this to the induction of the proteins necessary for glycan degradation. Furthermore, we connect the TCS07 regulon to virulence in a mouse model, thereby establishing that host-derived glycan-metabolism is important for infection *in vivo*. Finally, a comparative phylogenomic analysis of strains from the *Streptococcus* genus reveal that TCS07 and most of its regulon is specifically conserved in species that utilize host-glycans for growth.

## Author summary

Worldwide, *Streptococcus pneumoniae* is the most common cause of community acquired pneumonia with high mortality rates. Interestingly, *S*. *pneumoniae* strictly relies on carbohydrate scavenging for energy acquisition, which are obtained from the host. This is a critical step in pathogenesis and a common mechanism among Streptococcal species. In this study, we discover an uncharacterized two-component system that responds to the carbohydrate structures present on the host cells. These are important findings as we describe the molecular mechanism responsible for sensing these host derived glycans, and how this mechanism is linked to virulence, thus highlighting that glycan metabolism is important for infection *in vivo*, thereby posing a novel target for intervention. Our phylogenetic analysis reveals that the two-component system and the genetic regulon co-occur and are specifically conserved among Streptococcal species capable of degrading host-glycans.

## Introduction

Streptococci are facultative anaerobic Gram-positive bacteria and there are more than 100 different species in the genus. They frequently colonize the human upper respiratory -and urogenital tracts and are the predominant genus of the oral-cavity. Streptococci are usually non-invasive and does not harm their host. However, only *S*. *thermophilus* is considered entirely non-pathogenic as all remaining species have virulence genes and can cause disease when physical barriers are compromised or in an immunocompromised host.

One important aspect of their pathogenesis is carbohydrate acquisition. They strictly rely on carbohydrates for energy acquisition, due to the lack of the citric acid cycle. This is reflected in that Streptococci along with Clostridia and Enterococci are among the species with the highest abundance of carbohydrate transport systems relative to their genome size [1]. However, readily available carbohydrates are scarce in many niches of the human host which Streptococci colonize or invade. In these niches, some streptococci utilize host-glycan metabolism.

The model organism for glycan metabolism, *Streptococcus pneumoniae* (pneumococcus), is an opportunistic pathogen and a frequent colonizer of the human nasopharynx, a part of the upper respiratory tract. From the nasopharynx it can transverse the human epithelium and cause a wide range of infectious diseases such as otitis media, pneumonia, sepsis and meningitis [2]. *S*. *pneumoniae* is highly adapted to sensing and responding to specific signals resulting in the induction of a diverse set of virulence factors as well as DNA scavenging, adaptation to environmental stress, or adhesin and bacteriocin expression [3–8].

One mechanism by which *S*. *pneumoniae* coordinates gene expression is through two-component signal transduction systems (TCS). Combined, the components lead to a coordinated and differential expression of virulence factors necessary for successful disease progression [9]. Typically, the two-component systems consist of a membrane anchored histidine kinase (HK) and a cognate response regulator with a DNA binding motif (RR) [10]. The HK sensor protein is activated by an external stimulus resulting in autophosphorylation at a conserved histidine residue. The phosphoryl group is transferred to a conserved aspartate in the RR, which acts as a transcription factor that alters gene expression by binding to the promoter region of target genes [10].

Genomic studies have identified 13 putative TCSs and one orphan RR in *S*. *pneumoniae* [9,11]. One of the best characterised TCSs is the *comDE* operon, which controls genetic competence [12–13]. The HK ComD is activated by extracellular competence stimulating peptide (CSP), a quorum sensing signal that acts as an indicator for cell density [14–15]. The RR, ComE, then activates the expression of 24 early competence genes, including the alternative sigma factor ComX. Activation of the alternative sigma factor induces the expression of the late competence genes. The net result, among others, is the expression of proteins responsible for DNA uptake and recombination [4,14,16–17].

Several other TCSs of *S*. *pneumoniae* have been examined and linked to pathogenicity and direct control of virulence factors [9,18]. For instance, the two-component system TCS06 directly regulates the protective antigen, CbpA, and controls the expression of PspA, another major virulence factor [19–20].

In this study, we examined the two-component system TCS07 (SPD_0157-SPD_0158). We used next-generation RNA sequencing after ectopic expression of the response regulator RR07 to get a global understanding of the regulon. We find that TCS07 regulates genes encoding proteins important for host-glycan processing and transporters for the released glycans, as well as intracellular glycan catabolising enzymes. Consistent with this observation, we find that a mutant strain with a deletion of TCS07 is unable to grow on the model glycoprotein fetuin.

Like other species of streptococci, *S*. *pneumoniae* strictly relies on carbohydrate metabolism for energy acquisition, which must be obtained from the host or possibly other microbes present in the niche [21]. The bacterium possesses a true arsenal of proteins dedicated to glycan metabolism and transport. Indeed, several studies have linked *S*. *pneumoniae’s* ability to degrade various glycomoieties to virulence traits and *in vivo* fitness [22–26]. The degradation of both *N*- and *O*-linked glycoconjugated structures on proteins present on the host cells involves a complex biochemical pathway that is well described in pneumococcus. The current model for complex *N*-glycan degradation is initiated by neuraminidase A, encoded by the *nanA* gene, which hydrolyses the terminally located sialic acid. BgaA and StrH expressed on the pneumococcal cell surface sequentially cleave galactose and *N*-acetyl-D-glucosamine (GlcNAc) respectively, leaving a core of mannose_3_-GlcNAc_2_ (Man_3_GlcNAc_2_), which is cleaved from the glycoprotein by EndoD. The mannose-rich glycan core structure is transported to the cytosol and ultimately processed into individual monosaccharides [26–28].

Here we show that TCS07 responds to the glycoconjugated model protein fetuin (for details on glycomoities present on fetuin see [29]), but not the individual monosaccharides present in the glycan structure such as mannose, galactose, *N-*acetyl-D-glucosamine (GlcNAc), *N*-acetyl-D-galactosamine (GalNAc) or, *N*-Acetylneuraminic acid (sialic acid). Furthermore, we find a TCS07-dependent activation of most of the glycan metabolizing genes including *strH*, *endoD* and all 6 genes of the newly described *c*arbohydrate *p*rocessing *l*ocus (*CPL*). In agreement with previous studies on glycan metabolism and *in vivo* fitness, we show that a deletion of TCS07 decreases virulence in a mouse model. Finally, we expand our findings to other important pathogens of the Streptococcus genus with a bioinformatics approach. 584 strains with fully sequenced genomes from 17 different species were used in the analysis. Interestingly, we find that TCS07 and regulated genes are specifically conserved among Streptococcal species capable of utilizing host-glycans for growth. Lastly, we show that many genes of the regulon cluster around TCS07 in the genome in most of these species.

## Results

### Ectopic expression of RR07 leads to induction of glycan-metabolizing enzymes

**Fig 1:**
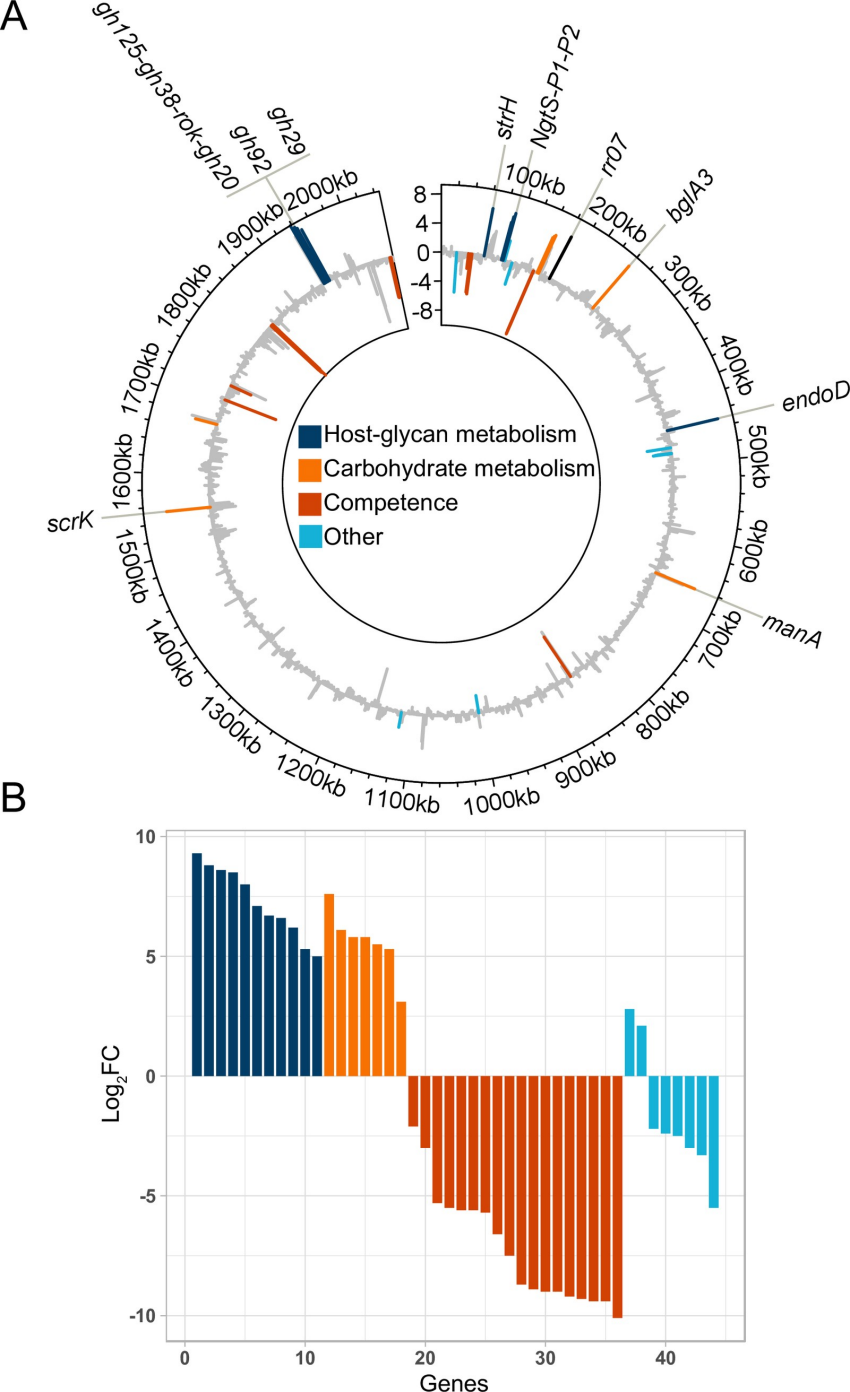
Overexpression of RR07 alters transcription of genes involved in host-glycan and carbohydrate metabolism. The transcriptome of wild-type D39 and the isogenic RR07 overexpressing mutant was compared by RNA-seq. Strains were grown in quadruplicates in C+Y to OD_600_ = 0.5 at which point cells were harvested and RNA extracted. The RNA was prepared for and sequenced as described in materials and methods. Sequence reads were mapped to the genome of D39, normalized and quantified. Log_2_ fold changes (Log_2_FC), false-discovery rates (FDR) and reads per million (RPM) were calculated for each gene. Genes were considered upregulated if Log_2_FC > 2, FDR < 0.05 and RPM > 500 in the RR07 overexpressing strain. Genes were considered downregulated if Log_2_FC < -2, FDR < 0.05 and RPM > 50 in the wild type strain. **A)** Circular plot of D39 genome with log_2_FC at y-axis and genomic location at x-axis. Up- or downregulated genes are color coded according to known or predicted functions (grey: genes not considered regulated based on above criteria). **B)** Log_2_FC of up- or downregulated genes sorted according to predicted or known function. 11 genes involved in host-glycan metabolism and 7 in carbohydrate metabolism are all upregulated.

We set out to examine the regulon of the two-component system TCS07 in *S*. *pneumoniae*. Previous studies have successfully overexpressed response regulators to identify TCS regulated genes [19,30]. We made a single-copy integration of the response regulator, RR07, downstream of a strong synthetic constitutive promoter at the versatile chromosomal expression platform (CEP) site in strain D39 [31]. The RR07-overexpressing strain and the corresponding isogenic wild-type strain were grown in rich C+Y medium to mid-exponential phase and total transcript abundance was measured by RNA-seq in quadruplicate. A total of 45 differentially expressed genes (Log_2_>2, FDR<0.05 and >50 or 500 RPM in the wild-type or RR07-overexpressing strains for down- or upregulated genes, respectively) were identified in the strain overexpressing RR07 (Fig 1A). The majority of the upregulated genes encode proteins involved in host-glycan and carbohydrate metabolism and transport (Fig 1B and Table 1).

**Table 1:** List of up- and downregulated genes when overexpressing RR07. Protein activities were attributed based on information retrieved from the KEGG and Uniprot databases as well as literature searches. See Fig 1 for criteria of up- and downregulated genes. ^a^names given for convenience in model illustration (Fig 9). ^a^our experiments suggest that the decrease of competence gene expression is due to an artefact from overexpression of RR07.

Locus tag	Name	Activity	Log2FC
Host glycan metabolism			
	**N-Glycan metabolism**		
SPD_0063	StrH	β-N-acetylhexosaminidase [32]	6.6
SPD_0088	NgtP1	ABC transporter permease [26]	5.3
SPD_0089	NgtP2	ABC transporter permease [26]	6.2
SPD_0090	NgtS	ABC transporter substrate-binding protein [26]	6.7
SPD_0444	EndoD (GH85)	*Endo*-β-N-acetylglucosaminidase [26][33]	7.1
SPD_1969	GH20	*Exo*-β-hexosaminidase [34]	9.3
SPD_1970	ROK	ROK family protein [26]	8.6
SPD_1971	GH38	Putative *exo*-α-1.3-mannosidase	8.8
SPD_1972	GH125	*Exo*-α-1.6-mannosidase [35]	8.5
SPD_1973	GH92	α-1.2-mannosidase [26]	5.0
SPD_1974	GH29	α-1.3/4-fucosidase [36]	8.0
**Carbohydrate metabolism**			
	**Glycosyl hydrolases and transferases**		
SPD_0138	GT1^a^	Glycosyl transferase family 1 protein	5.3
SPD_0139	GT2^a^	Glycosyl transferase family 2 protein	5.5
SPD_0140	ABC^a^	ABC transporter ATP-binding protein	5.8
SPD_0247	BglA3	Glycosyl hydrolase family protein [23]	7.6
	**Mannose and fructose metabolism**		
SPD_0641	ManA	Mannose-6-phosphate isomerase [37]	5.8
SPD_1531	ScrK	Fructokinase [38]	6.1
	**Starch and sucrose metabolism**		
SPD_1663	TreC	α.α-phosphotrehalase	3.1
**Competence**^**a**^			
	**Bacteriocin**		
SPD_0046	BlpU	Bacteriocin	-2.1
SPD_0132	-	Bacteriocin	-9.3
SPD_0133	-	Bacteriocin	-9.4
	**Competence**		
SPD_0049	ComA	Competence factor transporter [39]	-5.3
SPD_0050	ComB	Competence factor transporter [39]	-5.6
SPD_0844	ComEC	Competence protein ComEC [40]	-6.6
SPD_1711	SsbB	Single-strand DNA-binding protein [41]	-7.5
SPD_1740	CinA	Competence induced protein [42]	-3.0
SPD_1857	ComGG	Competence protein ComGG [40]	-8.9
SPD_1858	ComGF	Competence protein ComGF [40]	-9.4
SPD_1859	ComGE	Competence protein ComGE [40]	-8.7
SPD_1860	ComGD	Competence protein ComGD [40]	-9.2
SPD_1861	CglC	Competence protein CglC [43]	-9.0
SPD_1862	CglB	Competence protein CglB [43]	-10.1
SPD_1863	CglA	Competence protein CglA [43]	-9.0
SPD_2063	ComE	Competence response regulator [39]	-5.6
SPD_2064	ComD	Competence histidine kinase [39]	-5.7
SPD_2065	ComC1	Competence-stimulating peptide type 1 [39]	-5.5
**Other**			
	**Known**		
SPD_0994	RibF	Riboflavin biosynthesis protein	-2.4
SPD_1086	MutY	A/G-specific adenine glycosylase [44]	2.1
	**Unkown**		
SPD_0023	-	Hypothetical protein	-5.5
SPD_0092	-	Hypothetical protein	2.8
SPD_0104	-	LysM domain-containing protein	-3.0
SPD_0466	-	Hypothetical protein	-3.3
SPD_0474	-	Hypothetical protein	-2.2
SPD_0475	-	Uncharacterized protein	-2.5

We also analysed the promoter region of target genes, but we could not identify a consensus DNA binding motif for RR07. The transcriptomic datasets suggested that competence genes are downregulated by the expression of RR07. However, we consider these findings to be a result of RR07 overexpression artefacts and not genuine targets (see below).

**Fig 2:**
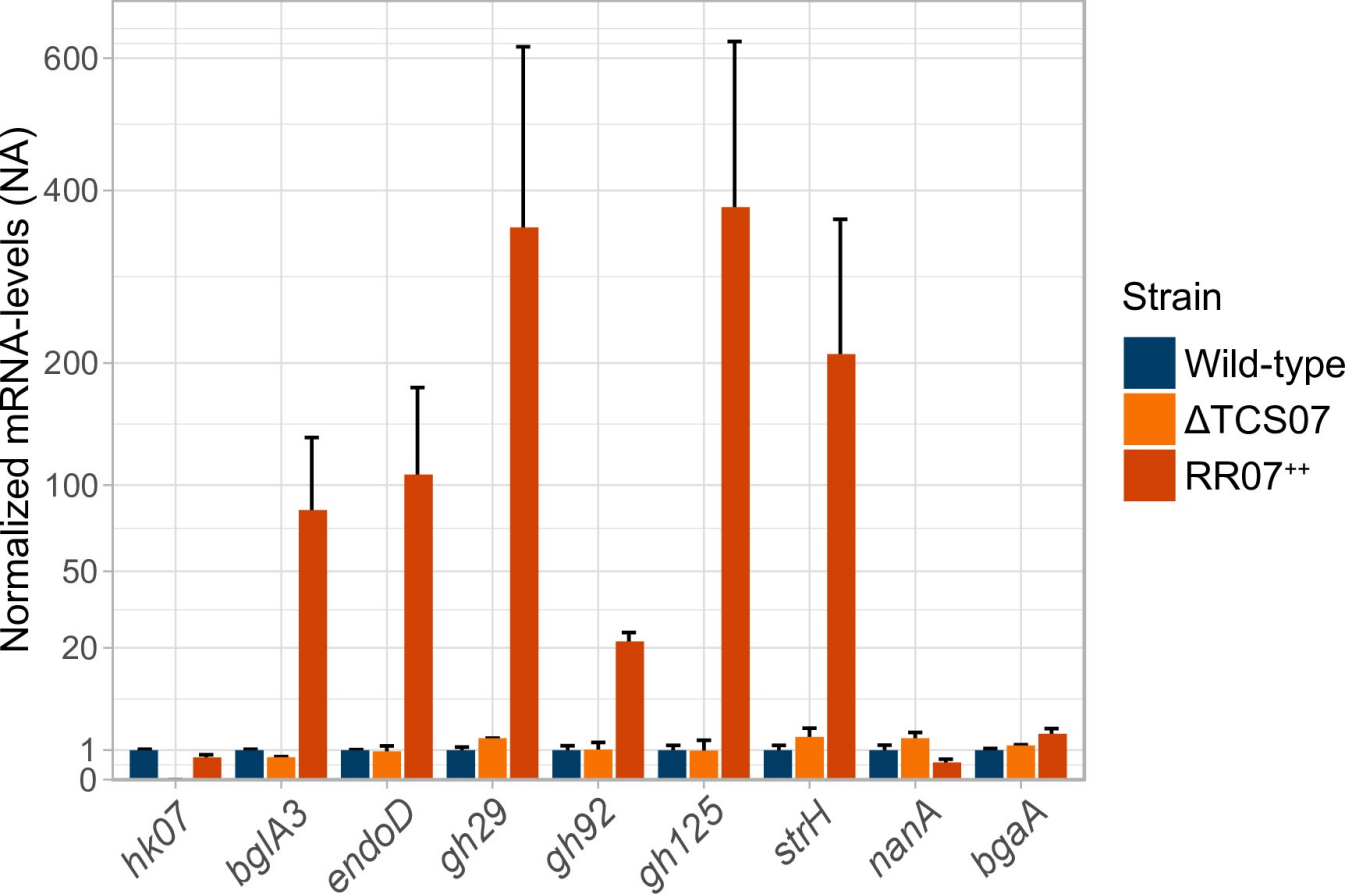
Validation of RNA-seq by RT-qPCR. Validation was performed by repeating the experimental setup for RNA-seq with inclusion of a *tcs07* mutant and performing RT-qPCR on the extracted RNA with primer sets for selected upregulated genes and *hk07*, *nanA* and *bgaA*. mRNA levels were normalized to *gyrA* and mRNA fold-changes are relative to the wild-type. Performed in biological duplicates with standard deviation as error bars.

To validate the induction of putative RR07 targets, we examined six candidate genes for further analysis by RT-qPCR. We included the genes encoding EndoD, BglA3 and StrH, as well as three genes encoding GH125 (based on our RNA-seq data GH125 is most likely in an operon with GH38, ROK and GH20), GH92 and, GH29, which are part of the recently described *C*arbohydrate *P*rocessing *L*ocus (CPL) [26]. We found that ectopic expression of RR07 resulted in a ~20-400-fold increase of all the selected candidate genes (Fig 2). In addition, we included the histidine kinase, HK07 in the analysis. We did not observe any induction of HK07, suggesting that RR07 does not regulate the transcription of its own promoter. Furthermore, we analysed the candidate genes in a strain deleted of the entire TCS07 operon. We found no changes in gene expression, suggesting that they are neither transcriptionally activated nor repressed by TCS07 in rich medium. Finally, we included *nanA* and *bgaA* in the analysis. NanA and BgaA are important enzymes in host-glycan metabolism as they cleave the terminally located sialic acid and galactose respectively from glycoconjugated proteins. We found no transcriptional activation of these genes by RT-qPCR analysis, confirming that *nanA* and *bgaA* are not induced by overexpression of RR07 (Fig 2).

### Growth characteristics of mutant TCS07

Our expression studies of RR07 suggested a link between TCS07 and carbohydrate metabolism.

**Fig 3:**
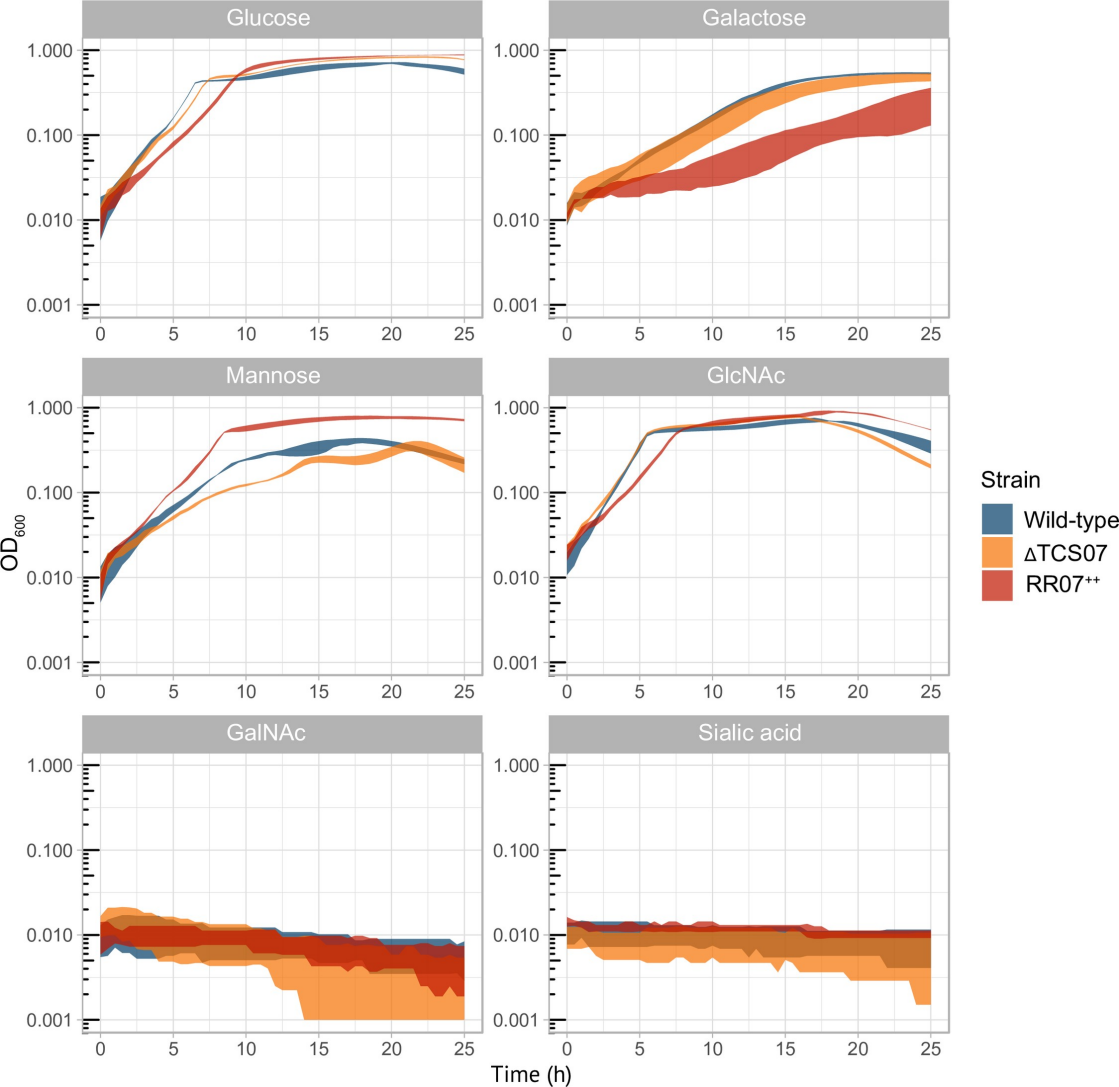
Growth experiments in glycan-derived sugars. Indicated strains were grown to exponential phase (OD_600_ = 0.3–0.4) in C+Y, at which point they were spun down and resuspended to OD_600_ = 0.1 in PBS. 20 μL was added to 180 μL CDM supplemented with 0.5% of the indicated sugar. Standard deviation depicted as colored-shadow. Performed in triplicates at least three independent times.

In order to determine the functional role of TCS07 we decided to examine the growth of a TCS07 deletion and a wild-type strain in a chemically defined minimum medium (CDM) supplemented with different relevant carbohydrates (Fig 3). We also included the RR07 overexpression strain for comparison. We found that the wild-type and TCS07 mutant grew similarly in glucose, galactose and GlcNAc minimal media. Conversely, the RR07 overexpression strain have decreased growth rates in these media (S1 Fig). However, in mannose minimal medium, the RR07 expressing strain was able to reach a higher cell density compared with the wild-type and TCS07 mutant strains (OD_600_ max for RR07 ~ 0.75 and OD max for wt ~ 0.4). This is consistent with a previous observation showing that ectopic expression of mannose 6-phosphate isomerase, encoded by *manA*, showed better growth than wild-type cells in mannose supplemented medium [45], since we observe that *manA* is part of the TCS07 regulon (Table 1). None of the strains were able to grow in medium supplemented with sialic acid or *N*-acetyl galactosamine (GalNAc), which have also been reported elsewhere for strain D39 [45–46]. However, other pneumococcal strains like TIGR4 can grow on sialic acid as a carbohydrate source [47–48].

### TCS07 is required for growth on the glycoprotein fetuin

**Fig 4:**
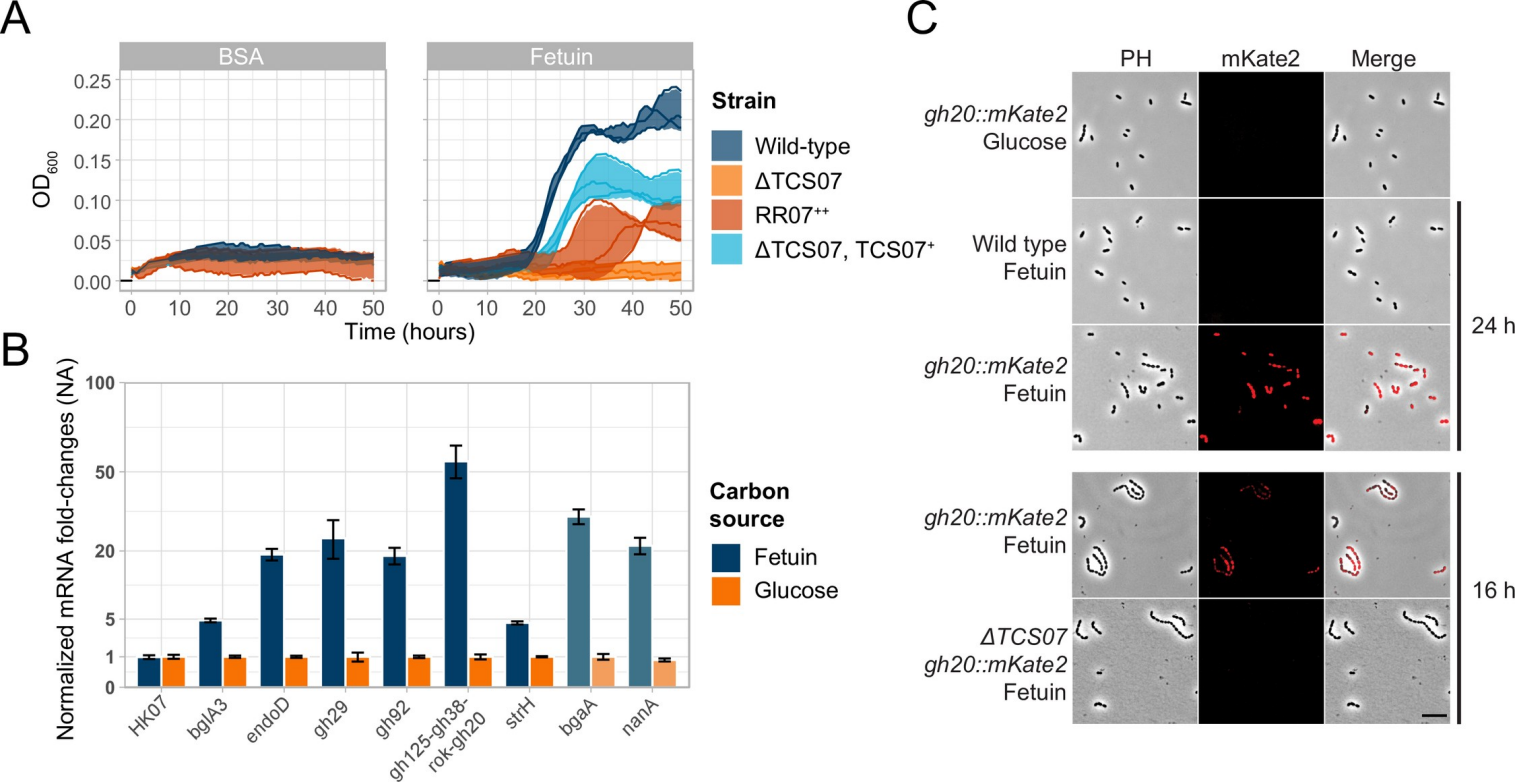
TCS07 is required for growth in and responding to a model glycan protein, fetuin. **A)** Growth experiments in fetuin (glycosylated) and BSA (not glycosylated). Indicated strains were grown to exponential phase (OD_600_ = 0.3–0.4) in C+Y, at which point they were spun down and resuspended to OD_600_ = 0.1 in PBS. 20 μL was added to 180 μL CDM supplemented with 2% fetuin or BSA. Lines depict replicates and colored shadows depict standard deviation. **B)** Growth in fetuin increase transcription of host-glycan metabolizing genes. D39 wild-type was grown to exponential phase (OD_600_ = 0.3–0.4) in C+Y, at which point the culture was spun down and resuspended to OD_600_ = 0.1 in PBS. 200 μL was added to 1800 μL CDM supplemented with 0.5% glucose or 2% fetuin. Cultures were grown to OD_600_ = 0.2 at which point cells were harvested and RNA extracted. RT-qPCR was performed on the extracted RNA with primer sets for indicated genes. mRNA levels were normalized to *gyrA* and mRNA fold-changes for each gene are relative to glucose. Faded genes (*nanA* and *bgaA*) is not part of the identified regulon of TCS07. Performed in biological duplicates with standard deviation as error bars. **C)** TCS07 is required for induction of the *gh125-gh38-ROK-gh20* operon. Fluorescence microscopy of wild type and ΔTCS07 strains with *gh20*::*mKate2* fusion. The wild type strain with the *gh20*::*mKate* fusion was incubated in CDM with 2% fetuin for 24 h (after growth initiated) or in CDM with 0.5% glucose until exponential phase. The wild type strain without the fusion was incubated in CDM with fetuin as a negative control. The wild type and ΔTCS07 with the *gh20*::*mKate2* fusion were incubated in CDM with 2% fetuin for 16 h (before growth initiated). Scale bar is 10 μm.

Many of the genes upregulated by ectopic expression of RR07 are involved in host glycan metabolism. We were inspired to test the growth of the TCS07 mutant strain in CDM medium supplemented with fetuin as the only carbon source. Fetuin is a model glycoprotein bearing complex *N*-linked glycan structures as well as *O*-linked glycans, which can be deglycosylated by *S*. *pneumoniae* [26,49–50]. The wild-type strain is capable of growth on fetuin alone, but the mutant was reproducibly unable to grow (Fig 4A). To verify this result, we made a fully complemented mutant strain by inserting the entire TCS07 operon at the CEP site in front of a constitutive promoter. This mutation could restore growth on fetuin, although it did not reach the same cell density as wild-type cells. We noted a long lag-phase, and optimal growth of wild-type cells was detected between 18–30 hours. In addition, we also examined growth in the presence of BSA, which does not contain any glycosylation sites (Fig 4A). None of the strains were able to grow in BSA, indicating that the growth phenotype on fetuin is attributable to the glycan moieties.

The fact that the TCS07 deletion strain is unable to support growth on fetuin encouraged us to look at the expression levels of the previously identified target genes by RT-qPCR. We grew wild-type cells in fetuin supplemented CDM and examined the expression of the target genes during exponential growth. All the candidates showed increased expression levels when grown in fetuin-supplemented medium compared to expression levels when grown in medium containing glucose (Fig 4B). In addition, we included *nanA* and *bgaA* in the analysis. They are not a part of the TCS07 regulon but we found that they are both induced in fetuin supplemented medium.

To substantiate and validate our finding, we decided to label one of the TCS07 target genes with a fluorescent tag. We made a transcriptional fusion of GH20, which is a part of the carbohydrate processing locus, with the far-red protein mKate2. We studied this fusion by fluorescence microscopy after 24 h incubation in fetuin supplemented CDM. We observed an intense fluorescence signal of the reporter strain in fetuin medium, but no florescence was detected when grown in glucose CDM (Fig 4C). We wanted to examine if this induction depended on TCS07. Since the TCS07 mutant was unable to grow on fetuin we were unable to perform RT-qPCR analysis. Instead, we used the GH20::mKate2 fusion construct as a representative of RR07 induced genes. We investigated cells by fluorescence microscopy after incubation for 16 h in fetuin. We chose 16 h as this was a time point before the wild-type cells grew on fetuin, thus, both wild-type and TCS07 deletion strains are characterized by a non-growth phenotype. We observed a clear fluorescence signal in wild-type cells but no signal in the TCS07 background, showing that induction of GH20 in fetuin depends on a functional TCS07 allele. Taken together, we conclude that the ability of *S*. *pneumoniae* D39 to grow on fetuin requires a functional TCS07 allele in support of TCS07-dependent activation of glycan metabolizing proteins.

We noticed a transcriptional decrease of several genes in the competence regulon by ectopic expression of RR07 (Fig 1). We performed RT-qPCR of the competence genes *comA* and *comD* during growth in fetuin where TCS07 is active. In this situation we observed an induction of competence genes (S3 Fig). Therefore, it is not likely that TCS07 is directly involved in competence regulation. The transcriptomic analysis does not reflect a downregulation of competence *per ser*, rather, the competence system fails to be induced in the RR07^++^ strain. We did therefore not pursue these observations further.

Next, we wanted to examine if any of the individual carbohydrates present in the glycan structure could act as a specific ligand for the sensor kinase HK07 to stimulate RR07 regulated genes. Possible ligands for TCS07 include the monosaccharides present in glycan structures—mannose, GlcNAc, GalNAc, sialic acid and galactose. We prepared minimal CDM medium with the individual sugars as carbon source. Since strain D39 is unable to grow on sialic acid or GalNAc alone (Fig 3), we supplemented these media with mannose in order to get sample material for RT-qPCR analysis. None of the individual monosaccharides were able to induce the expression of TCS07-regulated genes (S2 Fig). This suggests that the exact ligand for HK07 is a more complex carbohydrate structure than the monosaccharides. We included *nanA* and *bgaA* in the analysis. We found that *nanA* was induced in all media when glucose was absent while *bgaA* was induced in the presence of galactose. This may also explain the observation that they are both induced in fetuin as no glucose was present and galactose is released from fetuin in this medium. In support of this, previous studies found that *nanA* and *bgaA* are regulated by CcpA [51–52]. In addition, it was found that *nanA* is also induced by NanR in presence of sialic acid [53].

### TCS07 contributes modestly to virulence in a mouse model

**Fig 5:**
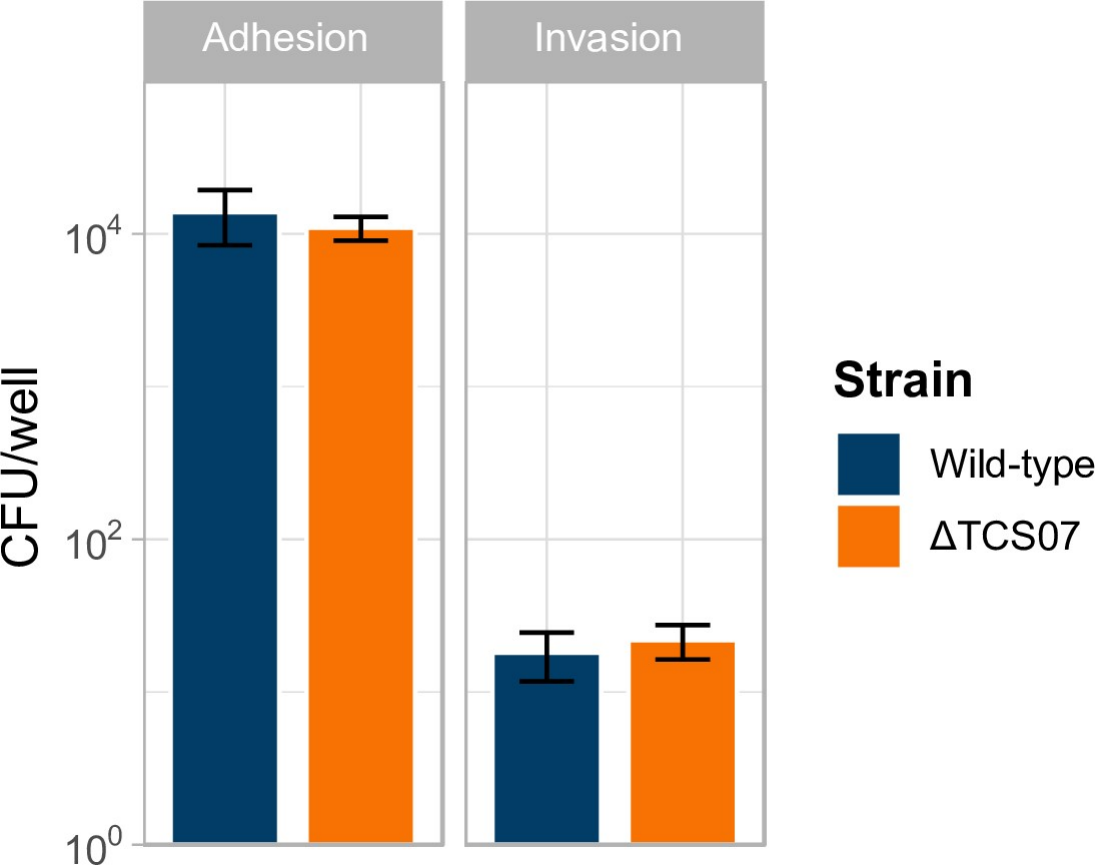
TCS07 does not affect adherence or invasion. Wild type and ΔTCS07 were inoculated from plate into DMEM to an OD_600_ = 0.2 and added to a confluent monolayer of A549 cells. Cells were incubated for 2 or 3 h for adherence or invasion assays, respectively. For adherence, non-adherent bacterial cells were washed away with PBS, and adherent cells were spread-plated to determine CFU counts. For invasion, non-invasive bacterial cells were killed by replacing the medium with DMEM supplemented with penicillin G and gentamycin and incubated for 1 h, before washing and plating to determine CFU counts. Adherence assays were performed in triplicates four individual times and invasion assays were performed in triplicates two times; error bars depicting standard deviation.

We sought to determine the role of TCS07 in pneumococcal colonization and virulence. First, we evaluated the ability of wild-type D39 and the isogenic TCS07 deletion mutant to adhere to and invade an *in vitro* model system using the human lung epithelial cell line A549. We found no significant difference in adherence or invasion between the wild-type and the mutant strain (Fig 5). Thus, TCS07 is neither important for adherence to, nor for invasion of, the human respiratory epithelium.

**Fig 6:**
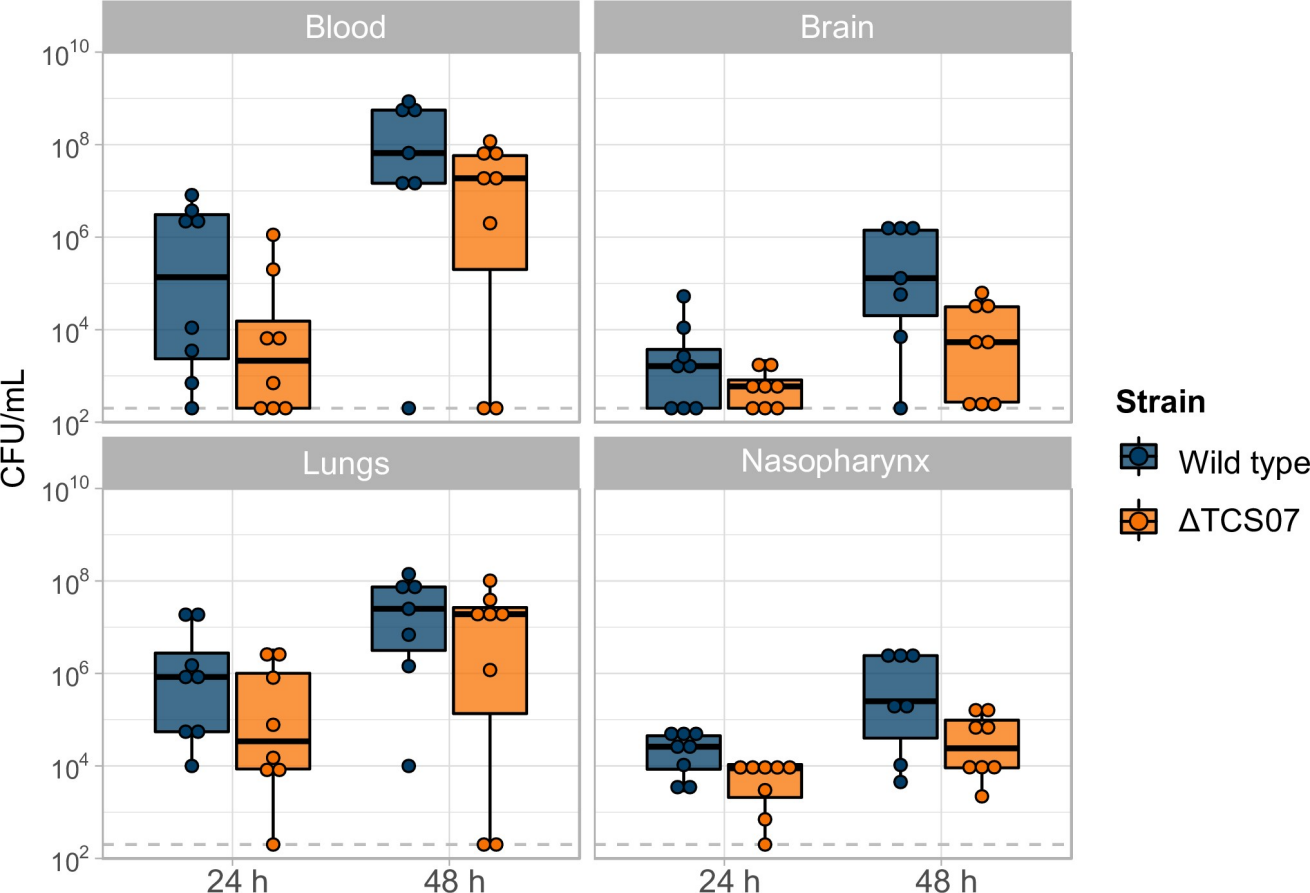
TCS07 increase virulence in a mouse infection model. 5 weeks old anesthetized female Swiss mice were challenged intranasally with 10^7^ CFU of wild type D39 or ΔTCS07. After 24 or 48 h mice were euthanised, blood and tissues were harvested and CFU was determined by serial dilution and plating. One mouse in the 48 h wild type group died from the infection. Statistical analysis was performed with a linear mixed effect model, (details in materials and methods). Deletion of TCS07 significantly reduced virulence (p-value = 0.02916, χ^2^(1) = 4.7579), and on average reduced bacterial loads 9.24-fold ± 2.67 (standard deviation).

We then assessed the contribution of TCS07 to virulence in a mouse model (Fig 6). Anesthetized mice (n = 8) were challenged intranasally with 10^7^ CFU of a D39 parent strain or the TCS07 mutant strain. We determined bacterial loads in different tissues at 24 h and 48 h post-infection. We used a linear mixed effect analysis of the relationship between bacterial load and strain. This statistical model is particular useful for this type of experiment because the model takes non-independent variables into account. Since multiple tissues were harvested from the same mouse, these measurements are non-independent. For instance, a mouse with relatively high bacterial load in the blood has a high likelihood of a relatively high bacterial load in the brain, which is indeed what we observed (S4 Fig).The model allowed us to investigate the overall virulence effect of the wild-type and TCS07 deletion strain across tissues and time points (see materials and methods). We observed a modest, but statistically significant decrease in virulence of the TCS07 mutant strain relative to the wild-type and the statistical model showed a lowered bacterial load (p-value = 0.02916, χ^2^(1) = 4.7579) across tissues and time points of mice with an average of 9.24-fold ± 2.67 (standard deviations). Combined, TCS07 has a modest effect on virulence in a mouse model and has no effect on adherence or invasion in *in vitro* assays.

### TCS07 and its regulon are conserved in glycan-metabolizing species

**Fig 7:**
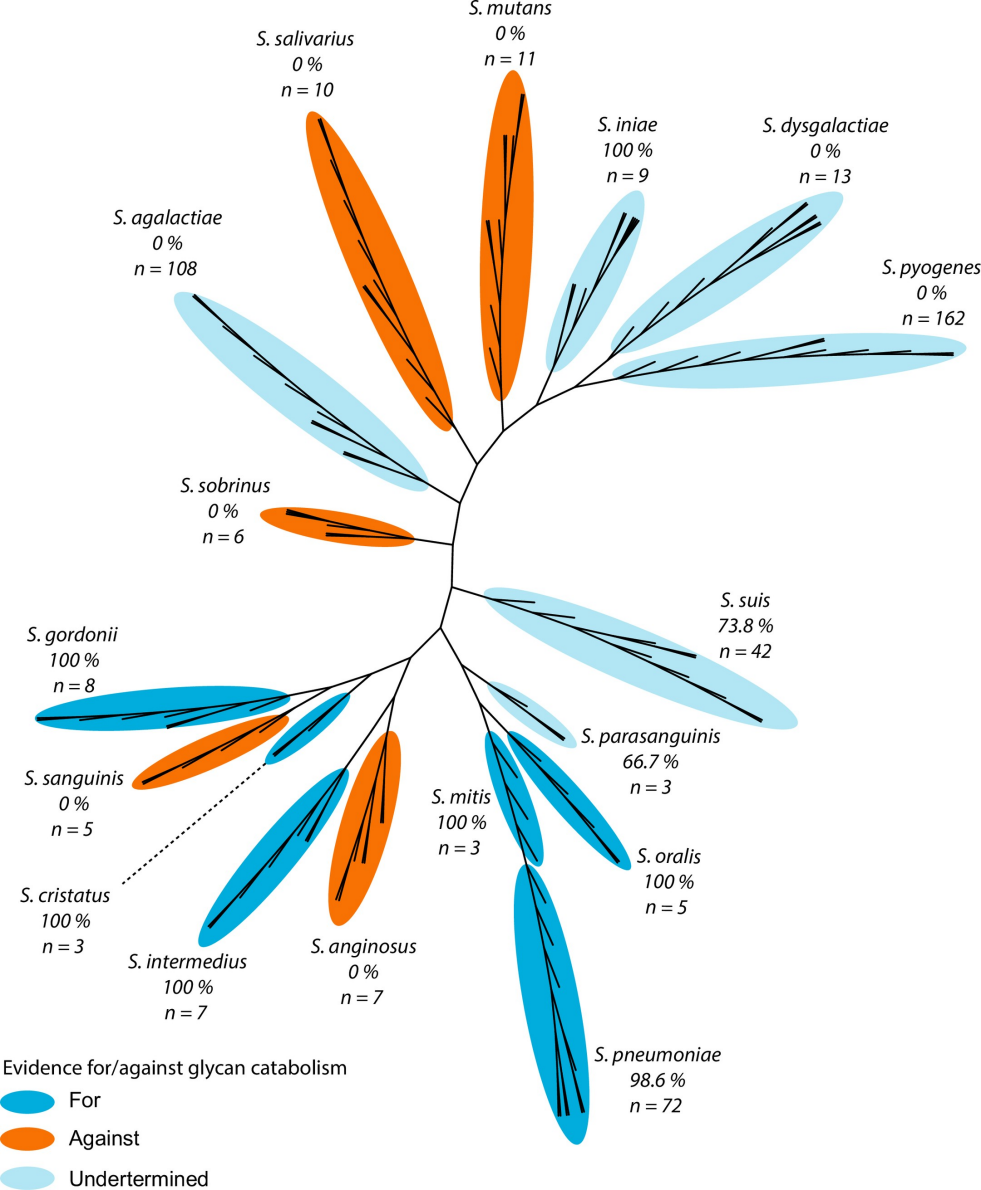
TCS07 is specifically enriched amongst species that catabolize glycans. Unrooted phylogenetic tree of important species from the *Streptococcus* genus. A maximum of 10 strains from each species were used for the phylogenetic tree. Numbers represent percentages of strains with a TCS07 homolog with the total number of strains from each species in parenthesis. All species with evidence for glycan catabolism have TCS07 homologs and all species with evidence against lack homologs. Evidence for or against is based on the literature (Table 2).

Host-glycan scavenging is exploited by many bacteria, including other species of streptococci [54–56]. However, the molecular mechanism for responding to and catabolizing glycans is unknown in most species. To expand the known mechanism from *S*. *pneumoniae* to species of the entire genus, we used a bioinformatics approach. First, the presence of TCS07 homologs within the genome was screened in all available complete genomes of streptococci. In almost all cases TCS07 is either completely absent or part of the core genome of individual species. Then, 17 relevant human pathogenic species were selected for detailed analysis, and an unrooted phylogenetic tree was created to visualise their relationships. Consulting previous studies of those species and glycan utilization, we find a clear correlation between evidence for glycan catabolism and the presence of a TCS07 homolog (Fig 7 and Table 2). Evidence for/against glycan catabolism is based on the ability/inability to grow on saliva or RNase B1 as well as the ability to modify these glycans, see references in Table 2.

**Table 2:** The presence of TCS07 in strains from different streptococci species correlates with known ability to catabolize glycans.

Species (n strains)	Main niche	Evidence^a^	TCS07^b^	References
*S*. *pneumoniae *(72)	Nasopharynx	For	98.6	
*S*. *cristatus *(3)	Oral cavity	For	100	[57][54]
*S*. *gordonii *(8)	Oral cavity	For	100	[57][54][58][59]
*S*. *iniae *(9)	Fish, meningens	-	100	[60]
*S*. *intermedius *(7)	Brain and liver	For	100	[54][61]
*S*. *mitis *(3)	Oral cavity	For	100	[57][54][58]
*S*. *oralis *(5)	Oral cavity	For	100	[57][54][56]
*S*. *suis *(42)	Swine	-	73.8	[62]
*S*. *parasanguinis *(3)	Oral cavity	For and against^c^	66.7	[54][58][59]
*S*. *agalactiae *(108)	Genital- and lower gastrointestinal tract	-	0	[63]
*S*. *anginosus *(7)	Genital- and lower gastrointestinal tract	Against	0	[54][61]
*S*. *dysgalactiae *(13)	Respiratory, gastrointestinal, and female genital tracts	-	0	[64]
*S*. *mutans *(11)	Oral cavity	Against	0	[54][65]
*S*. *pyogenes *(162)	Respiratory and lower gastrointestinal tracts, genital mucosa and skin	-	0	[66]
*S*. *salivarius *(10)	Oral cavity	Against	0	[54][58][59]
*S*. *sanguinis *(5)	Oral cavity	Against	0	[57][54][58][59]
*S*. *sobrinus *(6)	Oral cavity	Against	0	[59][65]

**Fig 8:**
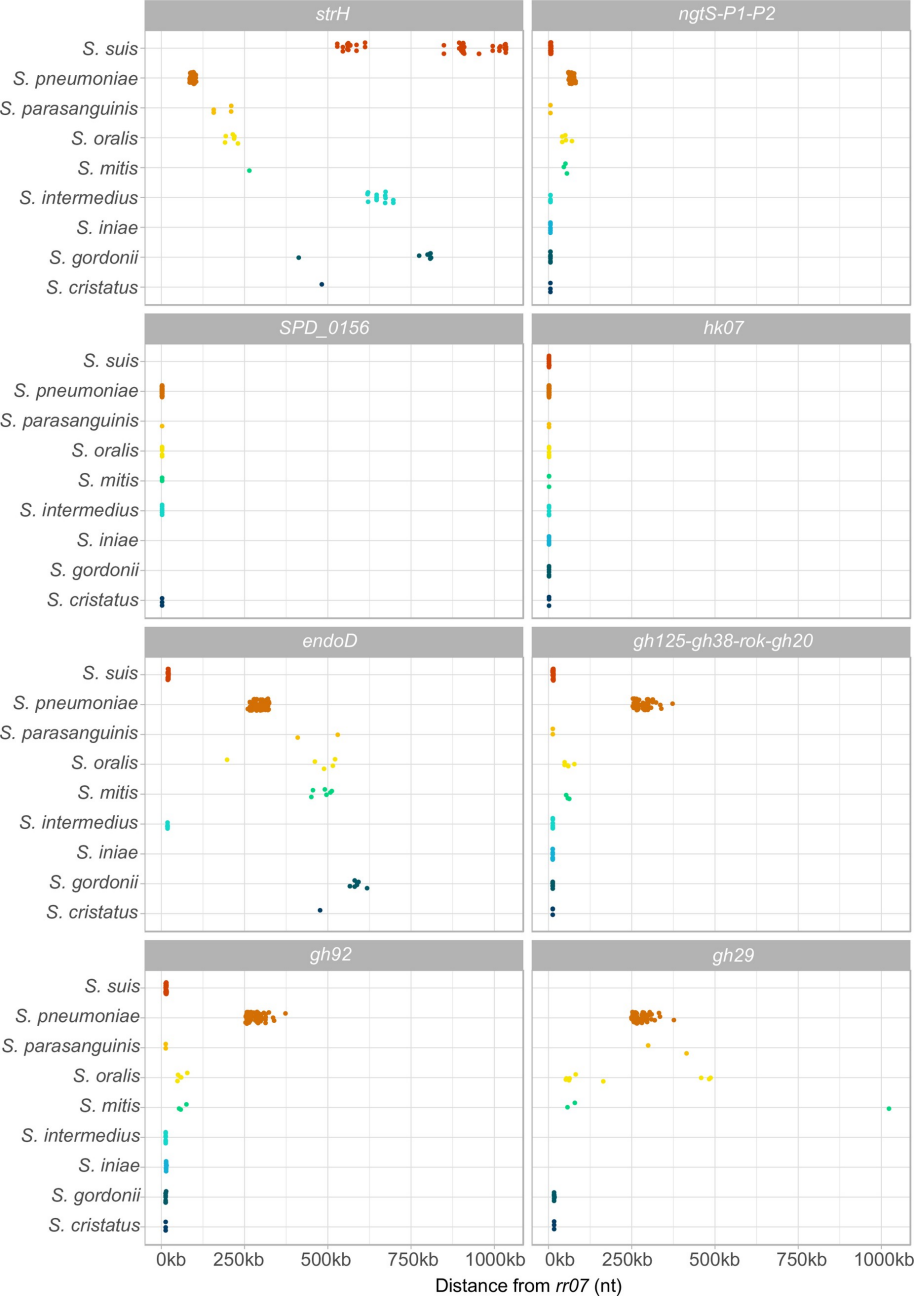
Most of the identified regulon clusters with TCS07 in the genome in other species of *Streptococci*. The genomic distance from the response regulator (*rr07*) to each gene of the regulon was determined in strains with a TCS07 homolog. For genes in operons, only the distance to the first gene was used, and each dot represents a gene homolog. The regulon is spread out in the genome of *S*. *pneumoniae*, whereas it mostly clusters around the TCS07 in other species. Except for *strH*, all genes cluster to TCS07 in at least two of the species. *SPD_0156*, a hypothetical protein, is part of the TCS07 operon in many species and otherwise absent. g*h29* is absent in three of the species. In two of them, *endoD* has replaced it genomic location, while it is absent in the last.

Since TCS07 is only present in some species, we sought to identify genes that co-occur with it (see materials and methods for details). Co-occurrence is the situation where two genes both tend to be present in the same genome [67]. Interestingly, most of the identified regulon in *S*. *pneumoniae* co-occur in other strains of streptococci carrying a TCS07 locus (Fig 8 and Table 3). Furthermore, while the regulon in *S*. *pneumoniae* is largely spread throughout the genome, the genes encoding the *CPL* locus, the ABC transporter and the TCS07 operon are co-localized in the genome of other species (*S*. *gordonii*, *S*. *cristatus*, *S*. *intermedius*, *S*. *parasanguinis*, *S*. *suis* and *S*. *iniae*). *gh29* of the *CPL* locus is missing in *S*. *intermedius*, *S*. *suis* and *S*. *iniae*. Instead, *endoD* has replaced it in its genomic location adjacent to *gh92* in *S*. *intermedius* and *S*. *suis*, while *endoD* has no homologs in *S*. *iniae*. *bglA3* is not specifically inherited with *tcs07*, but in *S*. *gordonii*, *S*. *cristatus* and *S*. *iniae* it is located just down-stream of *tcs07*. *strH* is absent in *S*. *iniae* and was otherwise not observed to co-localise with *tcs07*.

**Table 3:** Genes of the regulon are co-occur with TCS07. The presence or absence of each gene homolog was evaluated in 38 strains with TCS07 and 80 strains without TCS07. To prevent overrepresentation of strains from species with a high number of available genomes, a maximum of 3 strains were used from each species. The top 15 genes were chosen based on their p-value.

D39 locus	Gene name	Present with TCSO7	Present without TCS07	p-value
SPD_0157	HK07	38	0	7,99 x 10^−32^
SPD_1969	GH20	38	1	3,11 x 10^−30^
SPD_1970	ROK	38	1	3,11 x 10^−30^
SPD_0156	-	33	0	2,62 x 10^−24^
SPD_0444	EndoD	31	1	1,37 x 10^−20^
SPD_1973	GH92	38	14	1,41 x 10^−19^
SPD_0088	NgtP1	38	17	5,45 x 10^−18^
SPD_0090	NgtS	38	17	5,45 x 10^−18^
SPD_0158	RR07	38	17	5,45 x 10^−18^
SPD_1971	GH38	38	17	5,45 x 10^−18^
SPD_1972	GH125	38	17	5,45 x 10^−18^
SPD_0562	BgaA	28	1	1,18 x 10^−17^
SPD_0089	NgtP2	38	18	1,69 x 10^−17^
SPD_1803	TpiA	26	1	6,84 x 10^−16^
SPD_1974	GH29	29	4	1,36 x 10^−15^

Taken together, both experimental work and bioinformatics link TCS07 to host-glycan metabolism. Thus, the mechanism for sensing host-glycans and degrading them is likely conserved among other pathogens of the *Streptococcus* genus.

## Discussion

**Fig 9:**
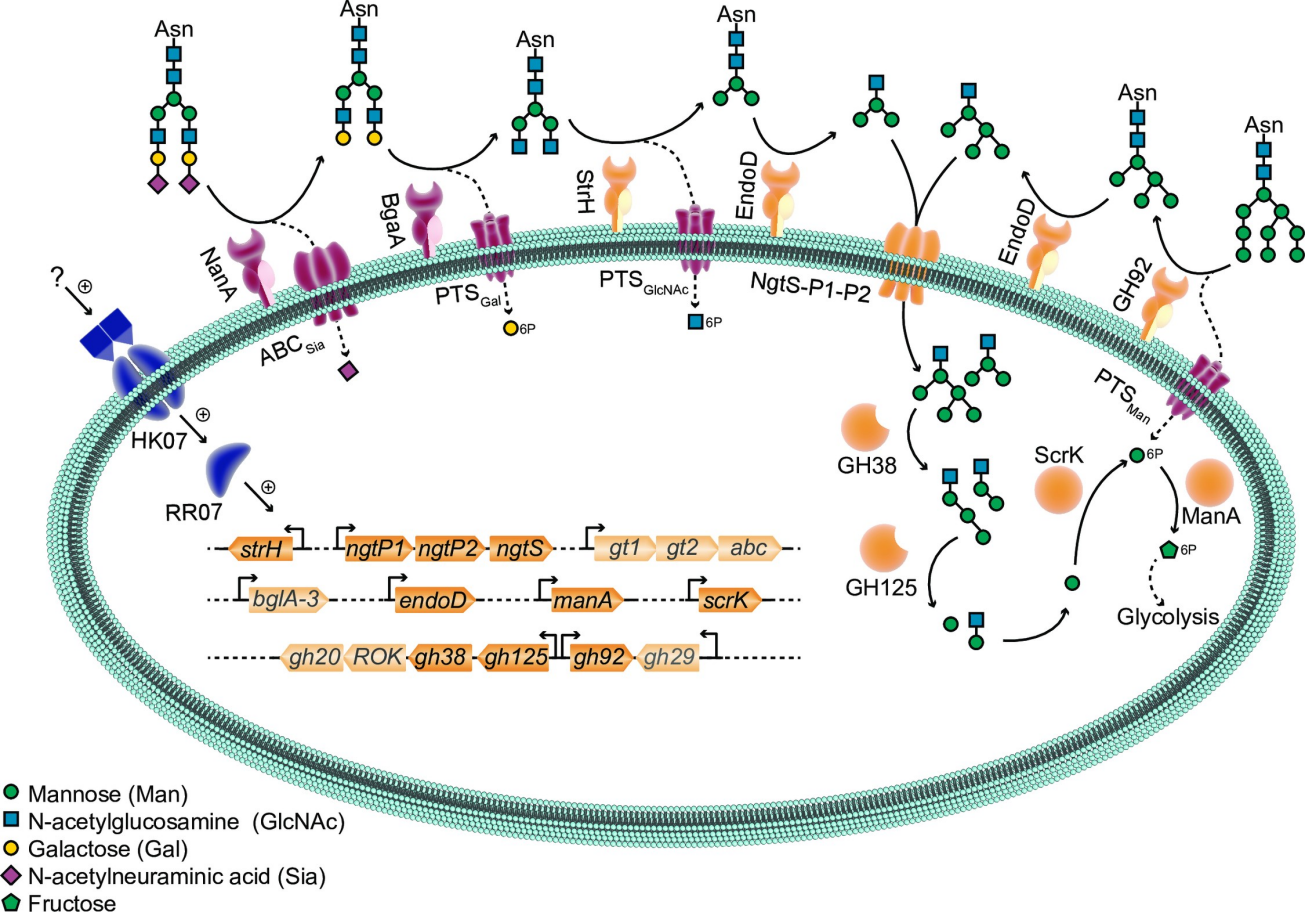
Model of *N*-glycan metabolism and TCS07 regulation. *N*-glycans are sequentially cleaved by extracellular and intracellular glycoside hydrolases, most of which are transcriptionally activated by TCS07 (HK07 and RR07). Genes upregulated by TCS07 and corresponding protein products are colored yellow. First step of degrading complex *N*-glycan structures is cleavage of sialic acid and galactose by NanA and BgaA, respectively, neither of which are part of the identified TCS07 regulon. All other enzymatic activities related to *N*-glycan metabolism can be attributed to protein products of TCS07 regulated genes as follows: GlcNAc exposed by NanA and BgaA is cleaved by StrH, and the resulting Man_3_GlcNAc_2_-core is cleaved between the GlcNAc moieties by EndoD. For high-mannose *N*-glycans, the Man_6-9_GlcNAc_2_ is trimmed by GH92 to Man_5_GlcNAc_2_, which is cleaved by EndoD to Man_5_GlcNAc. The released Man_3_GlcNAc and Man_5_GlcNAc are imported by the NgtS-P1-P2 ABC transporter system. Intracellularly GH38 hydrolyze the 1,3-glycoside bonds, and GH125 hydrolyze the 1,6-glycoside bonds of the Man_3_GlcNAc and Man_5_GlcNAc resulting in ManGlcNAc. Mannose released by GH38 and GH125 is possibly phosphorylated by ScrK into Mannose-6-phosphate, which is converted by ManA to fructose-6-phosphate. Finally, fructose-6-phosphate enters glycolysis. Genes that are faded are genes that has not been attributed a function in the model. Genes marked with * are genes that were not considered upregulated based on criteria defined in Fig 1 but are part of an operon with genes considered upregulated.

Tight control and coordinated induction of virulence genes by *S*. *pneumoniae* is likely to be important for colonization and disease progression. Here we studied an uncharacterised two-component system TCS07. This study provides new insight into how *S*. *pneumoniae* senses glycan moieties and regulates the transcriptome accordingly. To identify the potential targets of the response regulator, we performed RNA-seq analysis after ectopic expression of RR07 to get a global view of the regulon. We found that datasets overexpressing RR07 were highly enriched for genes encoding proteins responsible for host glycan and carbohydrate metabolism and transport (Fig 1 and Table 1). We have schematically summarized our finding in Fig 9. To complete the overview, we have included the expanded and proposed pathway for *N*-glycan metabolism by *S*. *pneumoniae* [26]. From the illustration, it is clear that TCS07 plays a major role in *N*-glycan metabolism by inducing many of the proteins in the *N*-glycan processing pathway. In fact, almost all proteins encoded by upregulated genes of the TCS07 regulon have known or predicted functions that fit in the model of sequential hydrolysis and transport of *N*-glycans and their derived carbohydrates. Ultimately, the sequential degradation leads to the conversion of mannose-6-phosphate into fructose-6-phosphate by ManA, which feeds directly into the glycolysis pathway (Fig 9).

The two initial deglycosylation steps in the process are removal of sialic acid by neuraminidase, NanA, and cleavage of galactose by β-galactosidase, BgaA. Interestingly, expression of RR07 did not result in the induction of these initiating enzymes. Host glycan metabolism, especially of *N*-glycans, is a major component of pneumococcal pathogenesis. It was suggested that the sequential deglycosylation by NanA, and BgaA reveals new receptor sites for adherence [28]. However, BgaA has a moonlight function since the β-galactosidase activity is not required for BgaA-mediated adherence [68]. Furthermore, BgaA binds directly to human epithelial cells supporting the hypothesis that BgaA acts as an adhesin [69]. *S*. *pneumoniae* relies on host glycans for energy scavenging and is able to sustain growth by utilizing the saccharides obtained from glycoproteins, thus suggesting a dual role of glycan metabolism in both adherence and energy production (Fig 4A) [70]. Interestingly, deletion of TCS07 had no effect on the adherence to, or invasion of, human lung epithelial cells (Fig 5), suggesting that induction of TCS07-regulated genes is not required for adherence. This is consistent with a previous observation showing that deletion of *strH*, which is a target of TCS07, does not contribute to adherence [28].

RNA-sequencing was used as an initial guide to identify putative target genes and thereby identify a possible physiological role of TCS07. We verified several upregulated targets *in vivo* (Fig 2). However, we do not consider all genes in the dataset as genuine targets. We were unable to identify any consensus binding motif for RR07, and further studies are needed to pinpoint the precise binding motif. Several genes were downregulated upon induction of RR07, suggesting that RR07 may also act as a negative regulator.

Interestingly, our mutant TCS07 strain was unable to grow on the model glycoprotein substrate fetuin (Fig 4). The growth-defective phenotype was only observed for fetuin as the TCS07 mutant showed no growth impairment in glucose, galactose, mannose or GlcNAc supplemented media (Fig 3). Sialic acid cannot be utilized by strain D39, and since BgaA is not a target of TCS07 we suspect that the released galactose is not sufficient to promote growth on fetuin.

At present, the specific ligand for TCS07 is unknown. TCS07 likely responds to *N*-linked glycans, but *O*-linked glycan moieties might also activate TCS07. Host proteins are also decorated with *O*-linked glycans, which can be deglycosylated by *S*. *penumoniae* [71]. However, *O*-linked glycans also contain terminal sialic acid and galactose, which are cleaved by NanA and BgaA respectively [50]. Neither NanA nor BgaA are part of the TCS07 regulon. Furthermore, the glycosidase Eng, which is also not part of the TCS07 regulon, is the only pneumococcal enzyme that exclusively degrades *O*-linked glycans. Finally, a previous micro-array study compared the transcriptome of D39 during growth on glucose with growth on mucin, which is a heavily *O*-glycosylated protein. They found that only *strH* of the TCS07 regulon was upregulated [45]. We therefore speculate that TCS07 responds specifically to *N*-glycan structures and we are currently investigating if that is indeed the case.

We found a TCS07-dependent activation of the fluorescence reporter strain GH20::mKate2 during growth in fetuin (Fig 4C). The exact stimulus for HK07 is present in fetuin (Fig 4), but it is not the monosaccharides of the glycan structure (S2 Fig), suggesting that TCS07 responds to a more complex carbohydrate structure. Currently we do not know if the glycan ligand for TCS07 must contain sialic acid or galactose. Since NanA and BgaA are not part of the TCS07 regulon, it is possible that the exact biochemical composition of the ligand is lacking these carbohydrates and may suggest that NanA and BgaA need to trim the terminal carbohydrates to reveal the ligand for TCS07 (Fig 9). Another possible ligand could be the mannose rich core structure (Man_3_GlcNAc or Man_5_GlcNAc) cleaved from fetuin by EndoD (Fig 9).

We examined the virulence characteristics of a TCS07 mutant strain. We observed a modest decrease in bacterial loads of the mutant allele across tissues and both time points. The minor decrease in virulence probably reflects that other carbohydrates are available *in vivo*. However, our finding is consistent with previous studies showing that a deletion of TCS07 in *S*. *pneumoniae* 0100993 (serotype 3) has a strong virulent influence in the lungs of mice [9]. In addition, EndoD and GH92, which both are part of the TCS07 regulon, contribute to virulence in a mouse model of pneumonia and sepsis in strain TIGR4 (serotype 4) [26]. Combined, these studies highlight the notion that glycan-metabolism is indeed important for infection *in vivo*.

Our phylogenetic analysis revealed that TCS07 and regulated genes are conserved among Streptococcal species that rely on carbohydrate savaging for energy acquisition (Fig 7 and Table 2). Scavenging glycans from host glycoproteins is also a common strategy in oral streptococcal species where they are able to survive using saliva as the only carbon source with a preference for *N*-linked glycans [55,72]. In addition, we found that the two-component system and its target genes cluster in the genome across many streptococcal species (Fig 8). A conspicuous feature of bacterial chromosomes is that genes encoding proteins in a common pathway are often clustered in distinct chromosomal locations [73]. These findings emphasize the notion that TCS07 is a major regulator of glycan scavenging.

*S*. *pneumoniae* most likely relies on glycan scavenging from host cells in a normal *in vivo* setting. It is therefore not surprising that the bacterium contains an arsenal of glycan and carbohydrate catabolizing enzymes and transporters. In this study we emphasized the role of TCS07 in glycan metabolism. However, glycan foraging in the pneumococcus-host interaction is much more nuanced and complicated than simply just nutrient acquisition, since modification of host glycans contribute to colonization and virulence in a variety of ways including biofilm formation, adherence and alteration of the immune response [27,74]. For instance, deletion mutants of NanA, BgaA or StrH exhibit increased C3 complement deposition and subsequent increased neutrophil killing [68,75].

Lastly, we also identified several genes of the TCS07 regulon of unknown function as putative targets for TCS07. Given the role in *N*-glycan metabolism of the target genes with known functions, these target genes with unknown functions are therefore promising candidates to examine for functional roles in host-glycan metabolism as well.

## Materials and methods

### Bacterial strains and growth conditions

All strains are listed in supplementary S1 Table in S1 Text. *Streptococcus pneumoniae* D39 and derivates were routinely grown in C+Y medium or on Columbia agar (Merck) supplemented with 2% (v/v) defibrillated horse-blood (SSI-Denmark) at 37°C with 5% CO_2_. Spectinomycin, kanamycin, or chloramphenicol were added at 100, 250 or 0.4 μg/mL, respectively, when appropriate. For growth and induction experiments, strains were grown in chemically defined medium (CDM) prepared as described previously with addition of 10 U/mL catalase (Merck) [76].

### Construction of genetically modified strains

All strains were genetically modified through direct transformation with linear DNA synthesized using AQ90 High Fidelity DNA polymerase (Ampliqon) or Phusion High-Fidelity DNA Polymerase (New England Biolabs). D39 was transformed in C+Y by addition of 0.2 μg/mL competence stimulating peptide 2 and purified PCR product. Transformed cells were plated on selection plates corresponding to acquired resistance. Colonies from all modified strains were sequence-verified by Sanger DNA sequencing. Detailed description of PCR synthesis is described in supplementary material.

### RNA extraction

RNA was extracted as described previously [77]. Briefly, cell pellets were resuspended in 150 μL cold solution 1 (10 mM Na-Citrate, 10 mM Na-acetate pH 4.5 and 2 mM EDTA) and added to a mixture of 150 μL solution 2 (10 mM Na-acetate pH 4.5 and 2% SDS), 700 μL acidic phenol (pH 4.5) and 300 μL chloroform. Tubes were inverted and heated to 80°C followed by brief cooling, and spin at 10,000 × *g* for 5 min. The aqueous phase was removed and precipitated in 96% ethanol. RNA was pelleted, washed in 70% ethanol and resuspended in H_2_O.

### RNA-sequencing experiment

D39 and ΔTCS07 were inoculated to an OD_600_ of 0.005 in C+Y in quadruplicates. Cultures were grown to OD_600_ of 0.5, at which point they were harvested. RNA was extracted as described above. 2.5 μg total RNA was depleted for ribosomal RNA with the Ribo-Zero rRNA Removal Kit for Gram-Positive Bacteria (Illumina). Subsequent library preparation was performed using the NEBNext RNA library prep kit for Illumina. Library quality was assessed using the Fragment Analyzer followed by library qPCR quantification (Kapa library quantification kit). Sequencing was carried out on a HiSeq1500 platform (Illumina). Raw-reads were mapped to D39 (assembly accession: GCA_000014375.1) using Bowtie2 [78] and quantified using the RNA-seq quantification pipeline in SeqMonk (Babraham Bioinformatic). Differential expression was calculated using the Bioconductor package *EdgeR* in R [79], and illustrative representation of data was created using the *Circlize* package [80]. Data are deposited in the GEO and SRA database with accession numbers GSE132733 and SRP201427, respectively.

### Reverse transcription quantitative PCR (RT-qPCR)

0.5 μg total RNA was DNase I treated (New England Biolabs), followed by heat inactivation of the DNase. cDNA was synthesized using the High-Capacity cDNA Reverse Transcription Kit (Applied Biosystems) according to manufacturer’s instructions. qPCR was performed in technical duplicates with 5 μL 2× RealQ Plus Master Mix Green (Ampliqon), 0.5 μL of each primer (10 μM; listed in S2 Table in S1 Text) and 4 μL diluted cDNA sample in a 384-well PCR plate (Thermo Scientific). The plate was run in a LightCycler 480 Instrument II (Roche). mRNA levels of target genes were normalized to *gyrA* mRNA levels. Results were analysed with the comparative critical threshold cycle method [81].

### Growth experiments

Strains were grown in C+Y to mid-exponential phase (OD_600_ = 0.3–0.4), at which point cultures were pelleted and resuspended in PBS. The OD was adjusted to OD_600_ = 0.1 in PBS, and 20 μL was added to 180 μL CDM with indicated carbon sources in a 96-well clear flat-bottomed Microplate (Falcon). The plate was incubated at 37°C with a gas permeable membrane (Breathe-Easy) in a Synergy H1 microplate reader (Biotek) and OD_600_ was measured every 30 min for the duration of growth.

### Induction experiments

D39 wild-type was grown in C+Y to mid-exponential phase (OD_600_ = 0.3–0.4), pelleted and resuspend in PBS. The OD was adjusted to OD_600_ = 0.1 in PBS, and 200 μL was added to 1800 μL CDM with indicated carbon sources. Cultures were grown to OD_600_ = 0.2 at which point samples were harvested

### Microscopy

D39 wild-type and GH20::mKate2 were grown as described under “Induction experiments”. For comparison of GH20::mKate2 and ΔTCS07 GH20::mKate2 induction in fetuin, cultures were adjusted to OD_600_ = 3.0 in PBS and incubated for 16 hours. At sampling points, 1 μL of culture was spotted on agarose pads (1% agarose, 0.9% (w/v) NaCl), and imaged on an inverted Olympus IX83 microscope with a Photometrics Prime sCOMS camera. Samples were excited with light at 580 nm and emitted light was captured through an FMCHE mirror cube (Olympus). Image processing was performed using Fiji [82].

### Adherence and invasion assays

The A549 lung epithelial cell line (ATCC) was cultured in DMEM high glucose (Gibco) supplemented with 100 μg/mL penicillin and streptomycin (PS) and 10% fetal bovine serum (FBS) at 37°C 5% CO_2_. Cells were detached from the bottom with non-enzymatic cell dissociation solution (Merck) to ensure glycan structures were not cleaved off. Detached cells were resuspended to approximately 10^5^ cells/mL and 1 mL was seeded into each well of a 24-well plate. Cells were grown to a confluent monolayer before they were washed in PBS, and re-incubated overnight in DMEM only supplemented with 1% FBS. For adherence assays, medium from wells with a confluent monolayer of A549 cells was exchanged with 1 mL DMEM 1% FBS with bacteria at OD_600_ = 0.2 (bacteria were scrapped off blood agar plates) and incubated for 2 hours at 37°C 5% CO_2_. Serial dilutions of bacterial inoculum were plated to ensure input of each strain was the same. After incubation, wells were washed thrice with PBS and cells were trypsinised with 100 μL 0.25% trypsin (Merck). 400 μL PBS 0.025% triton was added to disrupt eukaryotic cells. 25 μL samples were plated. Invasion assay was performed similarly, with few modifications: Infected monolayers were incubated for 3 hours, at which point the monolayer was washed with PBS. DMEM supplemented with 1% FBS, 10 μg/mL penicillin G and 200 μg/mL gentamycin was added for 1 hour to kill extracellular bacteria. After disruption of eukaryotic cells, 200 μL samples were plated.

### Animal infection model

6-week-old outbreed female Swiss mice (CD-1) were anesthetized by intraperitoneal injection with pentobarbital sodium (Nembutal; Rhone-Merieux) and challenged intranasally with 50 μL bacterial suspension in serum broth containing approximately 10^7^ cells. Challenge inoculum was verified by serial dilution and plating. Mice were euthanized by CO_2_ asphyxiation at 24 or 48 hours, and tissue samples (lungs, nasopharynx, brain, and blood) were harvested. Pneumococci were enumerated in tissue homogenates as described previously via serial dilution and plating on plates with 62 μg/mL gentamicin [83].

### Ethics Statement

This study was carried out in strict accordance with the recommendations in the Australian Code of Practice for the Care and Use of Animals for Scientific Purposes (8th Edition 2013) and the South Australian Animal Welfare Act 1985. The protocol was approved by the Animal Ethics Committee at The University of Adelaide (Approval number S-2016-183).

### Statistical analysis

Statistical analysis was performed using R and the *lme4* package to perform a linear mixed effect analysis of the relationship between bacterial load and strain. We used *strain*, *time* and *tissue* as fixed effects as they are independent measurements. For random effects we therefore used intercept for individual mice. Since multiple tissues were harvested from the same mice, these measurements are non-independent. The non-independence is thus resolved by assuming different baseline infection levels for each mouse. CFU/mL were log_10_-transformed to obtain normal distribution of measurements. We visually evaluated linearity, homoskedasticity and normality, and found no obvious deviation from the assumptions of the model. To check for absence of influential data, we used the *influence*.*ME* package to evaluate the influence of individual mice and found no major alteration of the result when excluding any mouse from the model. The likelihood ratio test was used to obtain a p-value. The test was performed by comparing the full model against a model excluding strain as fixed effect.

### Two-component system screening in Streptococcus

The presence of TCS07 in *Streptococcus* was screened on all complete genomes available (584 assemblies across more than 17 different *Streptococcus* species) from the National Center for Biotechnology Information, GenBank resource. Initially a tblastn search was performed with a cut-off E-value 10^-50, using the *rr07* and *hk07* sequences retrieved from strain 0100993. Hits were then sorted to only include strains with both an *rr07* and *hk07* hit within 1000bp of each other in the genome.

### Pangenome wide association study

All genomes retrieved were annotated using Prokka and a pan-genome was created using Roary [84,85]. The pan-genome was then analyzed using Scoary, predicting if any genes within the accessory genome were enriched amongst genomes containing the *tcs07* genes [86]. A trait file for Scoary was created using the presence absence matrix generated in the initial *tcs07* screening, reducing the amount of included strains of the same species to a maximum of 3, to minimize prediction bias of species-specific genes (118 assemblies). An unrooted phylogenetic tree of all the strains included in the analysis was generated by Roary using FastTree. Roary collects all the core genes that occur exactly once in each strain and uses it as an input for the phylogenetic analysis by FastTree, which then infers the phylogeny based upon SNPs (Single-Nucleotide Polymorphism) within these genes. The tree was visualized using iTOL v.4 interactive tree of life online tool [87].

The sensitivity of the pangenome clustering by Roary, was assessed by its ability to group known core genes in Streptococcus and was found to be sufficient in identifying most core genes at a 40% sequence identity cut-off. The sensitivity was also confirmed by the ability of Scoary to predict the *hk07* and *rr07* genes to strongly co-occur with *tcs07*.

## Supporting information

S1 Fig Doubling times. Indicated strains were grown to exponential phase in C+Y, at which point cultures were spun down and resuspended to OD600 = 0.1 in PBS. 20 μL was added to 180 μL CDM supplemented with 0.5% of the indicated sugars and OD600 was measured in a plate reader at 37°C. Doubling times were calculated during exponential growth. Grown in triplicates with 95% confidence intervals as error bars.
(TIF)

S2 Fig mRNA levels of TCS07 regulated genes in presence of glycan derived simple sugars. Wild-type D39 was grown to exponential phase in C+Y, at which point cultures were spun down and resuspended to OD600 = 0.1 in PBS. 200 μL was added to 1800 μL CDM supplemented with (A) 0.5% glucose, galactose, mannose, GlcNAc and (B) 0.25% mannose further supplemented with no extra, 0.5% GalNAc or Nacetylneuraminic acid (sialic acid). Cultures were grown to OD600 = 0.2 at which point cells were harvested and RNA extracted. RT-qPCR was performed on the extracted RNA with primer sets for indicated genes. mRNA levels were normalized to gyrA and mRNA fold-changes for each gene are relative to (A) glucose or (B) no extra. Performed in biological duplicates.
(TIF)

S3 Fig mRNA levels of comA and comD in fetuin. Grown and analyzed as described in Fig 4 in the main text. Performed in biological dublicates.
(TIF)

S4 Fig Bacterial load of each tissue at both time points of individual mice. (TIF)

S1 Text Supporting information text, tables and literature cited. (PDF)
