# Supplementary figures and images for: Host-glycan metabolism is regulated by a species-conserved two-component system in Streptococcus pneumoniae
Source: PLoS Pathog. 2020 Mar 4;16(3):e1008332. doi: 10.1371/journal.ppat.1008332 (PMC7075642; doi:10.1371/journal.ppat.1008332)

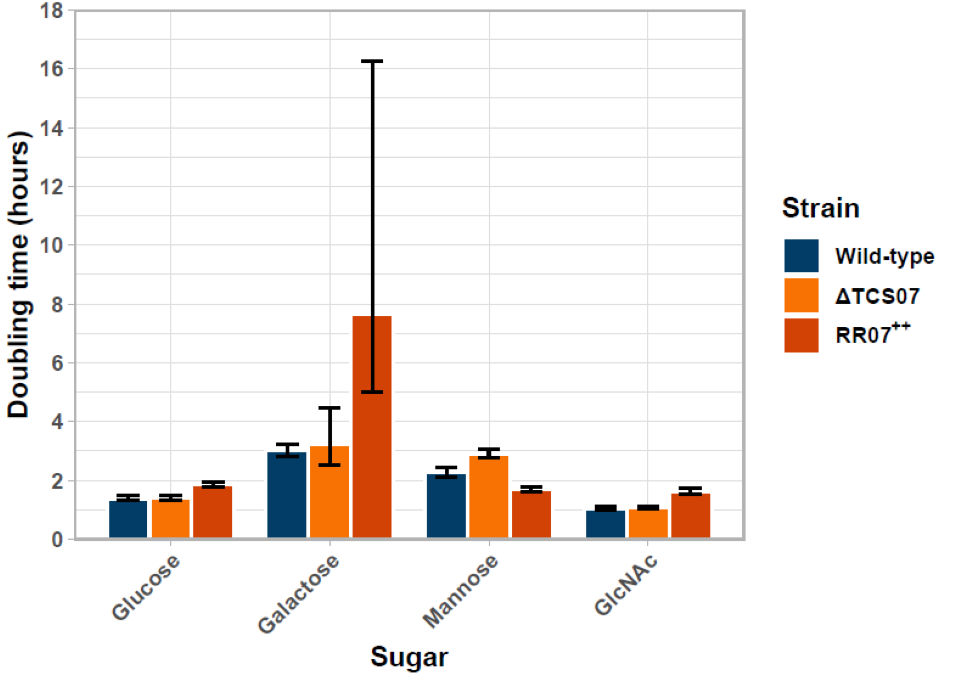

Supplement: S1 Fig — Indicated strains were grown to exponential phase in C+Y, at which point cultures were spun down and resuspended to OD600 = 0.1 in PBS. 20 μL was added to 180 μL CDM supplemented with 0.5% of the indicated sugars and OD600 was measured in a plate reader at 37°C. Doubling times were calculated during exponential growth. Grown in triplicates with 95% confidence intervals as error bars. (TIF) [file ppat.1008332.s001.tif]

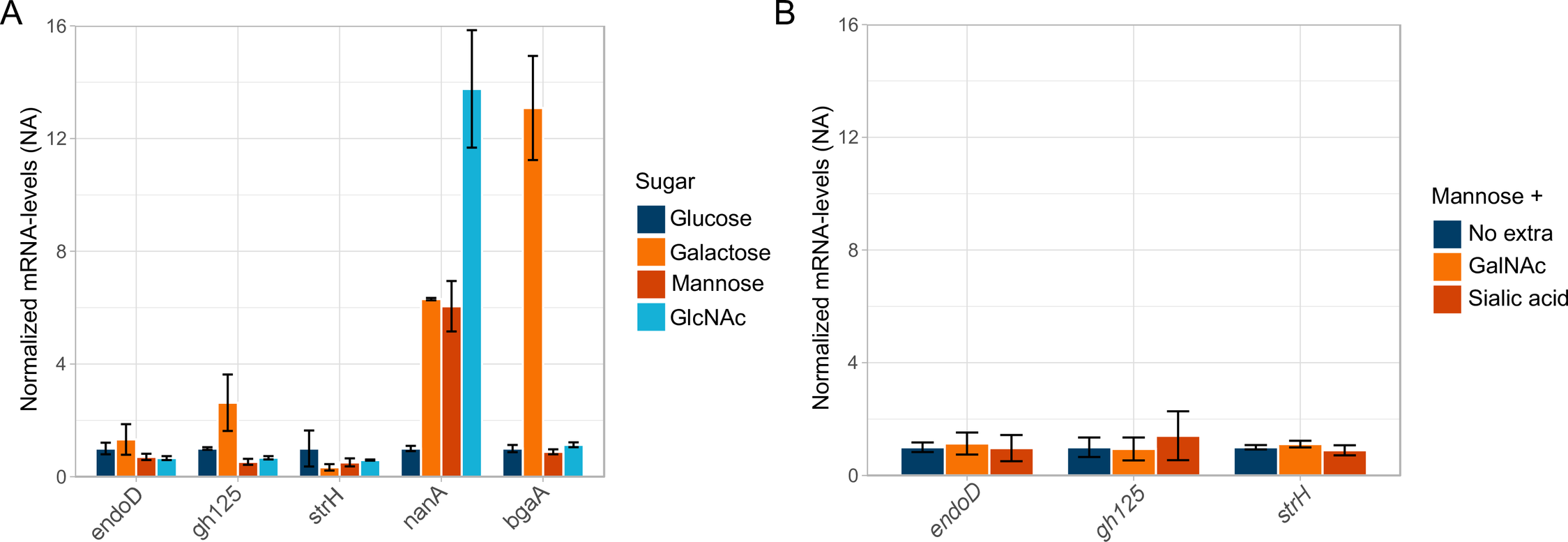

Supplement: S2 Fig — Wild-type D39 was grown to exponential phase in C+Y, at which point cultures were spun down and resuspended to OD600 = 0.1 in PBS. 200 μL was added to 1800 μL CDM supplemented with (A) 0.5% glucose, galactose, mannose, GlcNAc and (B) 0.25% mannose further supplemented with no extra, 0.5% GalNAc or Nacetylneuraminic acid (sialic acid). Cultures were grown to OD600 = 0.2 at which point cells were harvested and RNA extracted. RT-qPCR was performed on the extracted RNA with primer sets for indicated genes. mRNA levels were normalized to gyrA and mRNA fold-changes for each gene are relative to (A) glucose or (B) no extra. Performed in biological duplicates. (TIF) [file ppat.1008332.s002.tif]

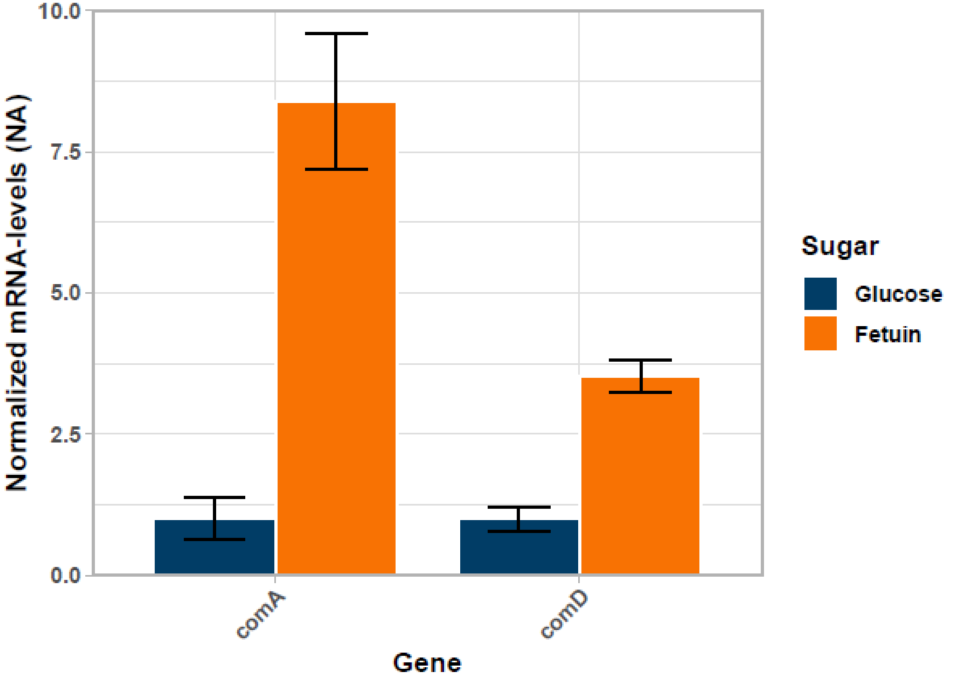

Supplement: S3 Fig — Grown and analyzed as described in Fig 4 in the main text. Performed in biological dublicates. (TIF) [file ppat.1008332.s003.tif]

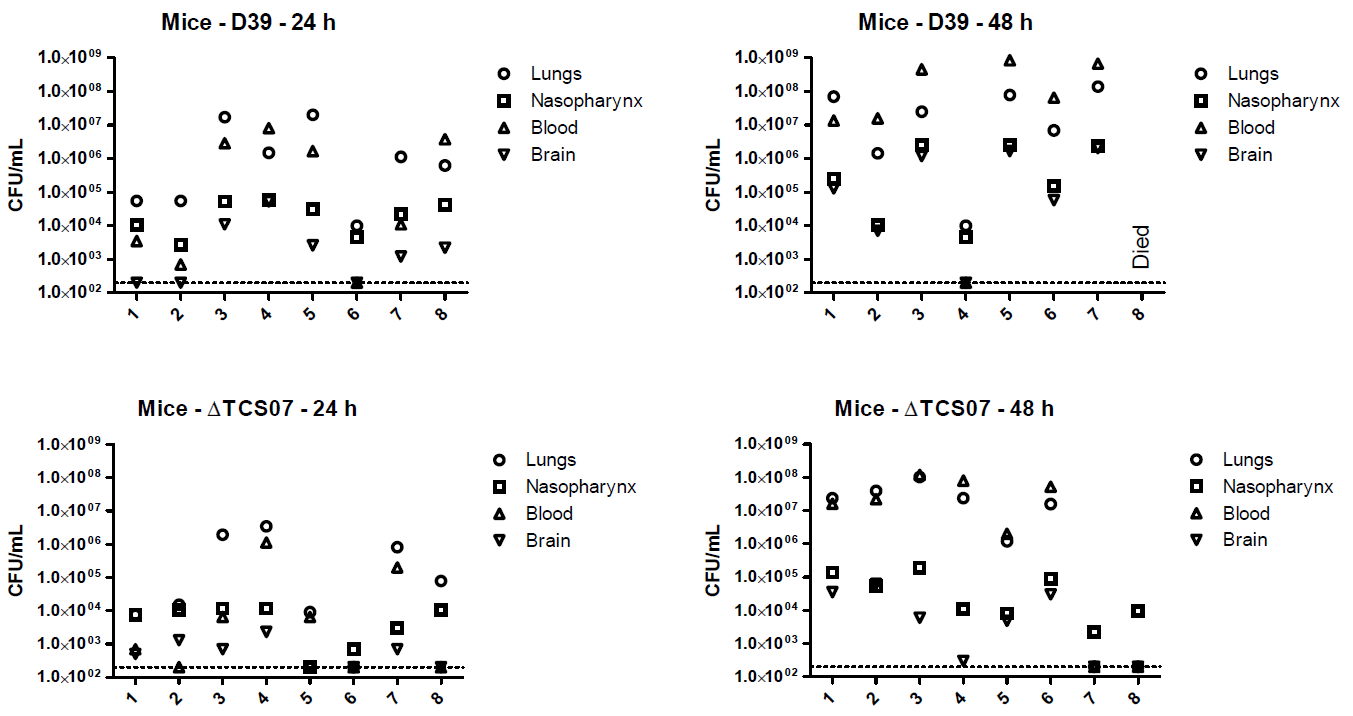

Supplement: S4 Fig — (TIF) [file ppat.1008332.s004.tif]
