# Supplementary material for: Host-glycan metabolism is regulated by a species-conserved two-component system in Streptococcus pneumoniae
Source: PLoS Pathog. 2020 Mar 4;16(3):e1008332. doi: 10.1371/journal.ppat.1008332 (PMC7075642; doi:10.1371/journal.ppat.1008332)
Supplement: S1 Text — (PDF) [file ppat.1008332.s005.pdf]

Text S1

Supporting information text, tables and literature cited

Host-glycan metabolism is regulated by a species-conserved two-component system in *Streptococcus pneumoniae*

Patrick Rosendahl Andreassen, Claudia Trappetti, Vikrant Minhas, Flemming Damgaard Nielsen, Kevin Pakula, James C. Paton & Mikkel Girke Jørgensen

Table S1: Strains used in the study

Strain	Genotype	Reference
**Wild-type**	D39 (serotype 2)	[1]
**RR07^{++}**	D39 *spe*^{R}::P_{con}::*SPD_0158*	This study
**ΔTCS07**	D39 Δ*SPD_0157-0158::kan*^{R}	This study
**ΔTCS07, TCS07$^+$**	ΔTCS07 *spe*^{R}::P_{con}::*SPD_0157-0158*	This study
**GH20::mKate2**	D39 *gh20::mKate2::*P_{con}::*cml*^{R}	This study
**ΔTCS07, GH20::mKate2**	ΔTCS07 *gh20::mKate2::*P_{con}::*cml*^{R}	This study

Table S2: Oligos used in the study

Name	Sequence (5' to 3')	Usage
**RR07_CEP_R**	CTCATCATCTACTAATAATACTTTATACATTAATTTTCCTCCT	**RR07^{++}**
**RR07_CEP_F**	CTACCGAAAACAGGTAGAAACTATACTATAATAAGGATCCCTCCAGTAACTCGAGA	
**RR07_F**	ATGTATAAAGTATTATTAGTAGATGATGAG	
**RR07_R**	TTATAGTATAGTTTCTACCTGTTTTCGGTAG	
**CEP_5'_F**	AGTTAAAGAACTTGGACTGCGC	**RR07^{++} & ΔTCS07, TCS07$^+$**
**CEP_3'_R**	CAATGTGAAAGCGATCAAGAACG	
**HK07_CEP_R**	CGTATGTAGCCAAAACTGCTTCATTAATTCCTCCTATTTAGATCTTGC	
**HK07_F**	ATGAAGCAGTTTTGGCTACATACG	
**HK07_1kb_F**	CCTTGTATTTTCCTAAATGAGCTACTCC	**ΔTCS07**
**HK07_kan_R**	GGATTATGGCCAATGAAGACTTTACTGTCTATTTTGTCGCCAATTTTTCAT	
**RR07_kan_F**	TTATATTTTACTGGATGAATTGTTTTAAGATTTGTATTCCTTTACAAAAGGTGCTA	
**RR07_1kb_R**	GGCAGTATTGGTTATTAAAGTTACGATTTCC	
**Kan_F**	CAGTAAAGTCTTCATTGGCCATA	
**Kan_R**	AAACAATTCATCCAGTAAAATATAATATTTTATTTTC	
**GH20_up_F**	GGAAACTCGTGTCTACCTAGAC	**GH20:: mKate2**
**GH20_up_R**	TAATTTTCCTCCTATTTAGATCTTGCATGCGAAGAGTATTAAGTCGTATAAATCG	
**mKate2_F**	CATGCAAGATCTAAATAGGAGGAAAATTA	
**mKate2_R**	GGGGACAGTGCAATGTCAAGTCTCGAGTTACTGGAGGGATCC	
**cml_R**	TTATAAAAGCCAGTCATTAGGCCTATC	
**pro_cml_F**	CTTGACATTGCACTGTCCCCCTGGTATAATAACTATACAGAGCACTAGTAGGAGGC ATATCAAATGA	
**GH20_down_F**	GATAGGCCTAATGACTGGCTTTTATAAAAAATCTCTTCAAACCACGTCAG	
**GH20_down_R**	CGTAAATCCAGCAAACTCAAGC	
**mKate2_R**	GGGGACAGTGCAATGTCAAGTCTCGAGTTACTGGAGGGATCC	
**hk07_qPCR_F**	CTCTTACGCTGACTGGGATTCAC	**qPCR**
**hk07_qPCR_R**	GTCTGGCATGCTAAGGCTAAAG	
**bglA3_qPCR_F**	CAGACAGAAGGACGTGTAGC	
**bglA3_qPCR_R**	CCGAAAGACTGTGTGACCAGTC	
**strH_qPCR_F**	GCTGTAGGAGCAGCTTCTGTTC	
**strH_qPCR_R**	AGCTTCTGGTTGAAGCTCTGC	
**gh125_qPCR_F**	TGGGTTGATGTCTTTGAGCATTG	
**gh125_qPCR_R**	ATGAAGGTAGGGTCTGAGTTGG	
**gh92_qPCR_F**	GCTTTGGTACTGCTAGCAAGC	
**gh92_qPCR_R**	GAAAGATAGGCAGATGCGGATC	
**gh29_qPCR_F**	AAGAAAATCAAACCGCATGGAC	
**gh29_qPCR_R**	GGGTTAAAGCGCTCAGGATCC	
**endoD_qPCR_F**	AAGTTATCAGTAGGAGCCTGCTC	
**endoD_qPCR_R**	GGTCGAATGCTCACTCTCTCC	
**gyrA_qPCR_F**	TGGTTGGACAGGTCTTGAGT	
**gyrA_qPCR_R**	TTTCACTCCCGTTCCTTGGA	

PCR strategy for strain construction

TCS07 was deleted with a PCR product consisting of a kanamycin resistance gene flanked by approximate 1 kb of the 5' and 3' flanking region of TCS07 (SPD_0157 and SPD_0158). The PCR product was constructed with a 2 step PCR: first, the 5' and 3' flanking regions and kanamycin resistance gene were amplified in individual reactions using primers HK07_1kb_F and HK07_kan_R, RR07_kan_F and RR07_1kb_R, and Kan_F and Kan_R, respectively. Subsequently, 0.1 ng/μL of each PCR product were used in the same reaction and the combined full-length PCR product was amplified with primers HK07_1kb_F and RR07_1kb_R. The PCR product was transformed into D39 and the resulting strain was named ΔTCS07.

RR07 was overexpressed using the chromosomal expression platform with a very strong constitutive promoter and consensus sequence Shine-Dalgarno as described previously [2][3]. Specifically, RR07 was overexpressed as described for D-PEP33, but with RR07 instead of GFP. The PCR product used for transformation was obtained in a 2 step PCR: first, the 5'-CEP region containing spectinomycin resistance, the 3' CEP region and RR07 gene were amplified in individual reaction using primers CEP_5'_F and RR07_CEP_R, RR07_CEP_F and CEP_3'_R, and RR07_F and RR07_R, respectively. Combined full-length PCR product was made as described above but with primers CEP_5'_F and CEP_3'_R. The PCR products was transformed into D39 and the resulting strain was named $RR07^{++}$.

TCS07 was complemented in a similar way as RR07 was overexpressed, except that 2 adenosine nucleotides were deleted between the Shine-Dalgarno and start codon to decrease translation rates. For this purpose, HK07_CEP_R and HK07_F were used instead of RR07_CEP_R and RR07_F, respectively. The PCR product was transformed into ΔTCS07 and the resulting strain was named ΔTCS07 $TCS07^{+}$.

GH20::mKate2 was constructed by incorporating *mKate2* with its own Shine-Dalgarno just downstream of GH20. A chloramphenicol resistance cassette with an independent constitutive promoter downstream of *mKate2* was used for selection. A PCR product consisting of *mKate2* and the chloramphenicol resistance cassette flanked by approximately 1 kb homology to the end of GH20 was synthesized in a 3 step PCR: First, the 5' flanking region, *mKate2,* the chloramphenicol resistance cassette and the 3' flanking region was synthesized with primers GH20_up_F and GH20_up_R, mKate2_F and mKate2_R, pro_cml_F and cml_R, and GH20_down_F and GH20_down_R, respectively. Then, the 5' flanking region and mKate2 was combined by mixing 0.1 ng/μL of each PCR product and using primers GH20_up_F and mKate2_R. Similarly, the chloramphenicol resistance cassette and the 3' flanking region was combined using primers pro_cml_F and GH20_down_R. Finally, the full-length PCR product was synthesized by mixing the 2 resulting PCR products and using primers GH20_up_F and GH20_down_R. The PCR product was transformed into D39 and ΔTCS07 and the resulting strains were named GH20::mKate2 and ΔTCS07 GH20::mKate2, respectively.

Programs

Prokka 1.13.3

Was used for predicting open reading frames and functional annotation of all genomes. This was done with the standard parameters of the software [2].

ncbi-blast+ 2.9.0

The tblastn function of the software was used for an in-house pipeline in screening for the presence of TCS07 in Streptococcus genomes.

The following input parameters were used: tblastn -subject ${fna_fil}bi -query $txt_fil -evalue 10E-50 -outfmt "6 "$fna_fil" qacc sseqid pident length gapopen qstart qend sstart send evalue bitscore slen " -out "$fna_fil".csv

Roary 3.12.0

Roary was used for creating the pangenome used as an input for the Scoary analysis as well as providing the input for the phylogenetic tree.

The following input parameters were used: roary -e --mafft -s -ap -cd 90.0 -p 24 -i 40 *.gff

Scoary 1.6.16

Scoary was used at default settings for predicting whether any genes in the accessory genome of the pangenome generated by Roary was co-inherited with a specified trait.

iTOL v.4

The iTOL interactive tree of life online tool was used for visualizing the phylogenetic tree generated by Roary [4].

Supplementary references

1. Lanie JA, Ng W-L, Kazmierczak KM, Andrzejewski TM, Davidsen TM, Wayne KJ, et al. Genome Sequence of Avery's Virulent Serotype 2 Strain D39 of *Streptococcus pneumoniae* and Comparison with That of Unencapsulated Laboratory Strain R6. J Bacteriol. 2007;189: 38–51. doi:10.1128/JB.01148-06

2. Guiral S, Hénard V, Laaberki M-H, Granadel C, Prudhomme M, Martin B, et al. Construction and evaluation of a chromosomal expression platform (CEP) for ectopic, maltose-driven gene expression in *Streptococcus pneumoniae*. Microbiology. 2006;152: 343–349. doi:10.1099/mic.0.28433-0

3. Sorg RA, Kuipers OP, Veening J-W. Gene Expression Platform for Synthetic Biology in the Human Pathogen *Streptococcus pneumoniae*. ACS Synth Biol. 2015;4: 228–239. doi:10.1021/sb500229s

4. Letunic I, Bork P. Interactive Tree Of Life (iTOL) v4: recent updates and new developments. Nucleic Acids Res. 2019;47: W256–W259. doi:10.1093/nar/gkz239
